# Effects of Creative Movement Therapies on Social Communication, Behavioral-Affective, Sensorimotor, Cognitive, and Functional Participation Skills of Individuals With Autism Spectrum Disorder: A Systematic Review

**DOI:** 10.3389/fpsyt.2021.722874

**Published:** 2021-11-18

**Authors:** Nidhi Amonkar, Wan-Chun Su, Anjana N. Bhat, Sudha M. Srinivasan

**Affiliations:** ^1^Physical Therapy Program, Department of Kinesiology, University of Connecticut, Storrs, CT, United States; ^2^Institute for Health, Intervention, and Policy, University of Connecticut, Storrs, CT, United States; ^3^The Connecticut Institute for the Brain and Cognitive Sciences, University of Connecticut, Storrs, CT, United States; ^4^Department of Physical Therapy, University of Delaware, Newark, DE, United States; ^5^Biomechanics and Movement Science Program, University of Delaware, Newark, DE, United States; ^6^Department of Psychological and Brain Sciences, University of Delaware, Newark, DE, United States

**Keywords:** creative movement, music, dance and movement, yoga, theater, martial arts, autism spectrum disorder (ASD), interventions

## Abstract

Autism Spectrum Disorder (ASD) is a neurodevelopmental disorder affecting multiple developmental domains including social communication, behavioral-affective, sensorimotor, and cognitive systems. There is growing evidence for the use of holistic, whole-body, Creative Movement Therapies (CMT) such as music, dance, yoga, theater, and martial arts in addressing the multisystem impairments in ASD. We conducted a comprehensive quantitative and qualitative review of the evidence to date on the effects of CMT on multiple systems in individuals with ASD. The strongest evidence, both in terms of quantity and quality, exists for music and martial arts-based interventions followed by yoga and theater, with very limited research on dance-based approaches. Our review of 72 studies (*N* = 1,939 participants) across participants with ASD ranging from 3 to 65 years of age suggests that at present there is consistent evidence from high quality studies for small-to-large sized improvements in social communication skills following music and martial arts therapies and medium-to-large improvements in motor and cognitive skills following yoga and martial arts training, with insufficient evidence to date for gains in affective, sensory, and functional participation domains following CMT. Although promising, our review serves as a call for more rigorous high-quality research to assess the multisystem effects of CMT in ASD. Based on the existing literature, we discuss implications of our findings for autism researchers and also provide evidence-based guidelines for clinicians to incorporate CMT approaches in their plan of care for individuals with ASD.

## Introduction

Autism Spectrum Disorder (ASD) is a neurodevelopmental disorder that affects multiple domains including the social communication, behavioral-affective, sensorimotor, and cognitive systems. Currently, around 1 in every 54 children in the United States qualify for a diagnosis of ASD (1) and boys are almost four times more likely to be diagnosed with ASD than girls of the same age (2). The hallmark impairments in ASD include poor reciprocal social interactions, difficulties with verbal and non-verbal communication, and restricted and repetitive behaviors and interests (3). For instance, children with ASD have difficulties in responding to social stimuli, sharing their play with peers and caregivers, developing and maintaining relationships, as well as understanding body language, gestures, and facial expressions of others (4–8). In terms of behavioral-affective impairments, children demonstrate repetitive and stereotyped behaviors such as finger flicking and hand flapping, highly circumscribed and restricted interests, insist on sameness relative to daily routines/schedules, demonstrate extreme distress to small changes in daily routines, and difficulties with transitions between activities (2, 9). Moreover, children may also have sensory symptoms including hypo- and hyper-sensitivity to sensory input and unusual responses to sensory stimuli in multiple domains including auditory, tactile-proprioceptive, vestibular, olfactory, and visual senses (9–12). In addition, children may also demonstrate disruptive behaviors such as aggression, tantrums, defiance, and self-injurious behaviors, as well as increased levels of negative affect (10, 11, 13, 14). Moreover, children with ASD also demonstrate cognitive difficulties such as attentional deficits, impaired decision-making, and impaired executive functioning (i.e., working memory, cognitive flexibility, self-control, generativity, and planning), with deficits being more pronounced during open-ended compared to structured settings (15–17).

Besides the diagnostic symptoms, children with ASD also exhibit a variety of other impairments within the sensorimotor domain that may lead to significant challenges in their activities of daily living (18–26). Although the exact prevalence estimates of motor impairments in ASD vary widely across studies from around 35% to over 85%, there is a growing consensus that children diagnosed with ASD exhibit motor impairments in gross and fine motor skills (e.g., bilateral coordination, gait and postural stability, handwriting, manual dexterity skills, and visuomotor control), as well as socially-embedded motor skills, including imitation, praxis (performance of skilled functional movement sequences/gestures), and interpersonal synchrony (ability to synchronize movements with those of another person) (19, 22, 27–38). Several studies have documented the association between motor impairments and severity of core autism symptoms in social communication, repetitive behaviors, and cognitive domains (22, 39–47). Moreover, sensorimotor difficulties could limit children's social participation and affect their activities of daily living including self-care, mobility, and leisure (41, 48–51). In short, children with ASD have multisystem impairments that need to be addressed through holistic evidence-based interventions (22, 24, 52–54).

Current standard interventions for ASD focus primarily on addressing the core social communication and behavioral impairments. Some popular evidence-based approaches include Applied Behavioral Analysis (ABA) (55), Treatment and Education of Autism and related Communication Handicapped Children (TEACHH) (56), Picture Exchange Communication System (PECS) (57), as well as developmental approaches such as Floor time (58), Social Communication, Emotional Regulation and Transactional Support Model (SCERTS) (59), Early Start Denver Model (ESDM) (60), and Pivotal Response Training (PRT) (61, 62). ABA-based approaches are considered the gold standard treatment for ASD and use principles of operant conditioning and intensive structured task practice to promote social communication and behavioral skills (55, 63–68). Similarly, the TEACHH approach uses visual cues to promote learning through picture schedules and also provides guidelines to increase structure and consistency in the environment, supplies-used, and therapists working with children with ASD (56, 69). Conventional therapies are usually very structured, adult-driven, and use a more sedentary approach (67, 70, 71). On the other hand, developmental approaches facilitate age-appropriate developmental skills such as joint attention, play, and imitation using child-preferred, play-based therapeutic activities within naturalistic settings (67, 70, 72). However, interestingly, both conventional and developmental approaches do not focus on addressing the sensorimotor impairments that are clearly highly prevalent in ASD (22, 46). This highlights a dire need to expand therapeutic interventions to address not just the core impairments but also the multiple co-morbidities in ASD.

Over the past several years, there has been a growing interest in exploring the effects of novel, alternative and integrated behavioral treatment approaches in addressing the multisystem impairments in ASD (27, 73–84). These holistic, whole-body movement-based, multisystem treatment approaches include but are not limited to structured physical activity, music therapies, yoga, martial arts, dance, and theater-based interventions (53, 73, 85–89). For the purpose of this review, we use the term “Creative Movement Therapy (CMT)” as an umbrella term that encompasses alternative behavioral interventions including music, dance, yoga, martial arts, and theater. The rationale for grouping these interventions together is that all these approaches use movement to integrate the social, emotional, cognitive, and physical aspects of the individual. Approaches involving CMT differ from conventional ASD interventions in that they are based in whole-body movement and promote self-expression (e.g., theater), creativity (e.g., innovative ways of moving body and using props in dance and theater), and improvisation (e.g., music making using instruments, moving to the rhythm of music). These interventions typically encourage child-led activities, playful exploration, and are therefore inherently more enjoyable and motivating for children with ASD (53, 90). From a theoretical perspective, CMT approaches are grounded in the ecological Dynamical Systems Theory (DST) (91, 92) and the Shared Affective Motion Experience (SAME) theory (93). The DST emphasizes that basic perception-action cycles of bodily movement form the basis for higher-order social communication and cognitive skills (82). Similarly, the SAME theory suggests that music- and movement-based experiences are multimodal in nature and activate similar “mirror” networks in the brain of participants, thereby forming the basis for social, emotional, and motoric connectedness between them (83). This is especially crucial for individuals with ASD given their deficits in multimodal integration stemming from long-distance brain under-connectivity (94–96).

In addition, given their very nature, CMT interventions are known to have multisystem effects on the sensorimotor domains as well as on the social communication, cognitive/attentional, and behavioral/affective domains in individuals with ASD. For instance, practicing simple and complex movement sequences during choreographed dance routines provides opportunities to promote rhythmic synchronization, multi-limb coordination, balance, gait, and postural control in participants (85). On the other hand, music-based group activities provide a medium for children with ASD to connect with social partners, improve communication abilities, and lead to greater positive affect/engagement (4, 6, 53, 93, 97–103). Similarly, short bouts of exercise that incorporate self-discipline, goal-oriented behavior, multistep action sequences, and sustained focus, as seen with any martial arts-based techniques, could enhance cognitive abilities such as executive functioning in children with ASD (104).

Although the preliminary evidence is promising, currently, it is unclear if CMT approaches can be considered as evidence-based interventions in ASD. Therefore, this review aims to synthesize the literature to date on the effects of CMT on social communication, behavioral-affective, cognitive, sensorimotor, and functional/participation skills of individuals with ASD across the lifespan (note that for the purpose of the review, we excluded studies that focused on structured physical activity, animal-assisted therapies, or technology-based interventions given the clear differences in the key intervention components of CMT approaches compared to the above-mentioned approaches). A few previous reviews have assessed the effects of CMT in children with ASD (75, 76, 78, 105). However, most of them have been restricted to examining the effects of a single type of CMT in individuals with ASD. It would be crucial to compile information on different CMT approaches to compare and contrast the differential effects of these approaches on multiple systems in ASD. Moreover, except a couple of reviews by Zou et al. and Geretsegger et al., none of the other reviews conducted a risk of bias analysis for the reviewed studies or calculated effect size (ES) estimates based on data reported in the reviewed literature (75, 105). Assessing methodological quality of studies through a risk of bias analysis enables researchers to estimate the level of confidence in study findings and guides interpretation of study results. Similarly, ES estimates from individual studies indicate the magnitude of the treatment effect and are thus crucial to evaluate the clinical utility of specific treatment approaches. We address these gaps in the literature by providing a comprehensive review of empirical reports studying the effects of CMT approaches through August 2021 in children with ASD. Specifically, we (i) summarize the narrative literature and compare the efficacy of different types of CMT in addressing multisystem impairments in individuals with ASD, and (ii) provide quantitative ES estimates for outcome measures addressed using CMT approaches to objectively evaluate the clinical importance of CMT for individuals with ASD.

## Methods

### Search Protocol

We reviewed literature from four different electronic databases related to allied health, psychology, physical therapy/kinesiology, and education, namely, PubMed (1950–2021), PsycINFO (1969–2021), Scopus (1966–2021), and CINAHL (1937–2021). The combination of key terms used included, (a) “*music*,” “*dance*,” “*yoga*,” and “*play*,” (b) “*intervention*,” “*therapy*,” and (c) “*autism*” (please see Appendix 1 for details of search strategy). We also conducted additional hand searches of reference sections of included studies and previous review papers to identify missed literature through August 2021.

### Eligibility Criteria

We included studies published in peer-reviewed journals that assessed the effects of creative movement and play-based therapies in individuals with ASD using experimental or quasi-experimental, longitudinal study designs. Studies were excluded based on the following criteria: (a) only included individuals with other developmental disabilities such as Cerebral Palsy, Down's Syndrome, Attention Deficit Hyperactivity Disorder, Intellectual disability, Spina Bifida, Dyslexia, Learning Disability, etc. [note that studies (*N* = 5) that recruited mixed samples i.e., individuals with ASD and individuals with other developmental diagnoses were included since we wanted the review to be comprehensive and inclusive of all studies that recruited samples of individuals with ASD], (b) review papers, case-studies, qualitative studies, purely narrative reports, observational studies or reports describing the protocol for a future study, (c) interventions directed solely toward parents/primary caregivers of individuals with ASD, (d) studies that used structured physical activity, animal-assisted therapies, or technology-based interventions in ASD, (e) reports in languages other than English, and (f) gray literature including theses and dissertations.

### Data Extraction and Evaluation

**Figure 1:**
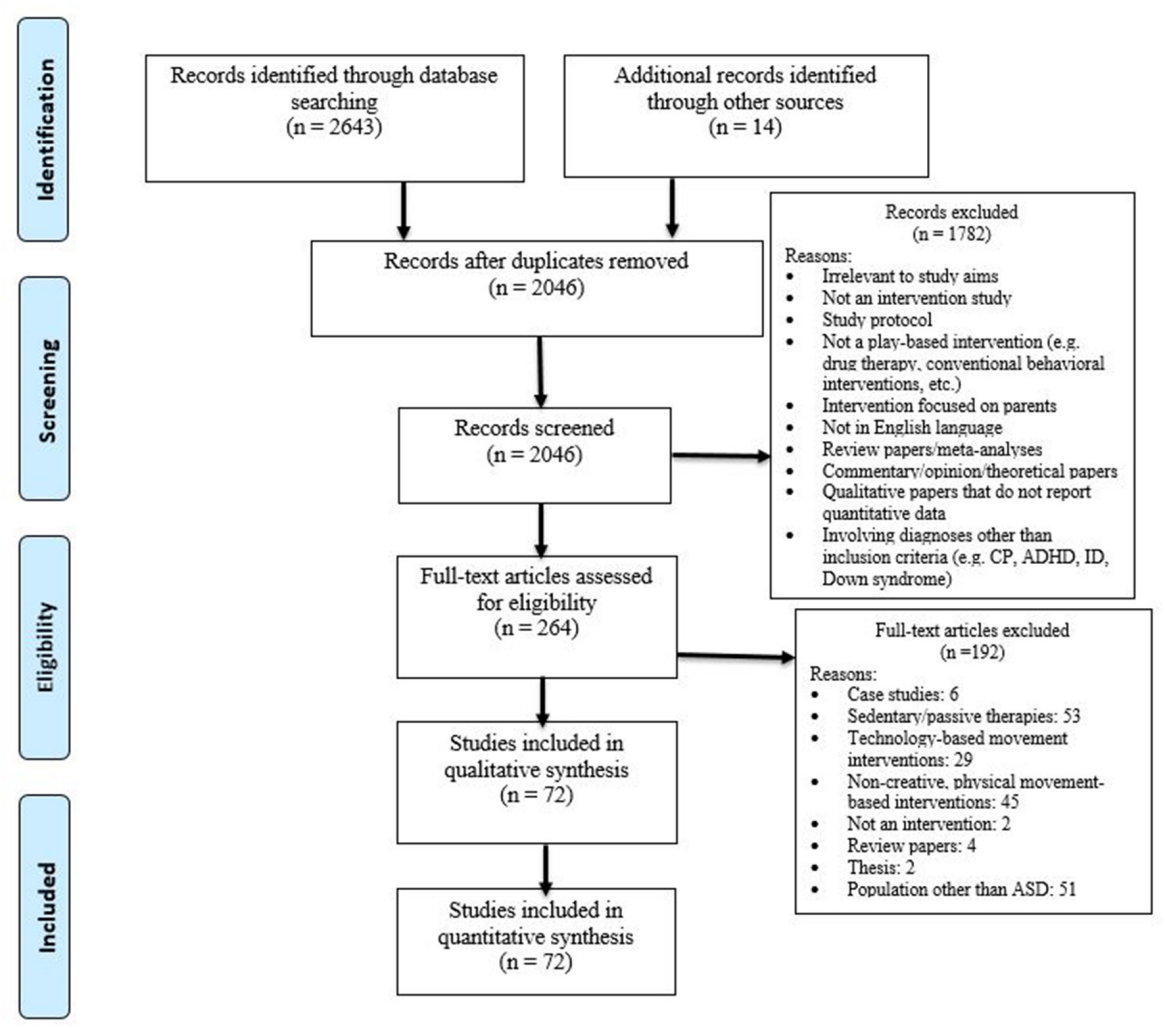
PRISMA flow diagram of search strategy and study selection process.

After screening 2,643 articles using our eligibility criteria [PubMed (1,354), PsycINFO (821), Scopus (267), and CINHAL (201)] and removing duplicates, 72 articles qualified for our review. Two trained research assistants and the last author screened titles and abstracts of the 2,643 articles based on our eligibility criteria. When necessary, full texts of articles were reviewed to assess eligibility of the study (see Figure 1 for details of search process). All three coders agreed in their ratings for 90% of studies. Disagreements between coders for study inclusion were resolved through discussions and consensus scoring.

#### Risk of Bias Assessment

We employed the Physiotherapy Evidence Database (PEDro) scale and the NIH quality assessment tool (106, 107) to assess risk of bias in reviewed studies. The PEDro scale was used to evaluate the internal and external validity of randomized controlled trials (RCTs) and controlled clinical trials (CCT's) included within our review. The PEDro has a total of 11 items which are scored on a dichotomous scale (No = 0, Yes = 1) of which 10 items are scored for each RCT/CCT to obtain a study score out of a maximum possible score of 10 (first item on the PEDro is not included in the total score) (106). Studies with a PEDro score ≥ 6 are classified as having low risk of bias. For single group pre-post designs, we used the NIH quality assessment tool to assess risk of bias (107). The NIH tool comprises 12 items that are scored on a dichotomous scale (No = 0, 1 = Yes) to assess internal validity of reviewed studies. Questions 6, 7, 9, and 10 include multiple questions per item. For these questions, if studies satisfied all criteria listed in the item, we gave them full points (score of 1). However, if studies satisfied some but not all criteria, a partial score of 0.5 was awarded for the item. The original tool recommends raters to categorize studies based on their risk of bias into categories of “good,” “fair,” and “poor” with studies rated as “good” having low risk of bias and studies rated as “poor” having high risk of bias (106). We classified studies with total scores ≥ 9 as “good,” studies with total scores ≤ 6 as “poor,” and all other studies as having “fair” quality. In addition to the above-mentioned tools, we also used the Levels of evidence as outlined by Sackett et al. (108) to classify all the 72 studies. This grading, based on study design, ranges from Levels I-V. We only included studies from Levels I up to III in our review. Level I is the highest level of evidence and includes systematic reviews, meta-analyses, and RCT's with a PEDro score of ≥ 6, Level II includes RCT's with a PEDro score < 6 and all CCT's, whereas Level III includes single group before-after (pre-post) study designs.

### Study Coding Procedures

We coded each study in the review for sample and study characteristics, methodological quality, intervention characteristics (*FITT*: Frequency, Intensity, *T*ime, *T*ype), assessments used, dependent variables, and treatment effects (see Appendix 2 for coding details). In addition to a narrative description of studies, we also report on quantitative ES from reviewed studies along with their confidence intervals to obtain estimates of the true magnitude of treatment effects following CMT in individuals with ASD. For parametric data, when adequate data were provided in the original report, we calculated ES i.e., standardized mean difference (*d*) values (109–111). For papers that reported non-parametric statistics, ES were calculated using *U*- and *z*- statistics (112). In studies where the original report did not provide estimates of central tendency and variability of measured outcomes, we calculated ES using parameter estimates (*F-* and *t*-values) and *p*-values. We acknowledge that these estimates are more inaccurate compared to ES estimates calculated using measures of central tendency and spread within the sample (see **Tables 4A–C** for details); however, we wanted to provide readers with ballpark estimates of ES. We classified ES according to Cohen's conventions as small (0.1–0.3), medium (0.3–0.49), or large (0.5 and above) (113). We also report 95% confidence intervals (CI) around ES estimates to identify robust, statistically significant effects of CMT in ASD (114, 115). Specifically, if a CI does not include 0, it implies a truly significant non-zero treatment effect at the 5% significance level. For the purpose of reliability, all authors as well as 2 undergraduate students coded a subset of the 72 studies using a detailed coding form. Intra-rater reliability of over 99% and inter-rater reliability of over 90% were achieved through consensus coding on scores that coders disagreed on. Following reliability, rest of the papers were divided and coded by the first and last authors.

## Results

### Description of Studies

All 72 studies reviewed were published between 1994 and 2021 although only 25% of the studies specifically mentioned the year of data collection in the published report. Out of the 72 studies that we reviewed, 25 used music therapy approaches, 11 studies employed yoga-based interventions, 16 studies assessed the efficacy of martial arts-based interventions, 12 studies employed theater-based interventions, 7 studies assessed the effects of dance, and lastly, 1 study employed a combination of music and dance therapies. Of these studies, 30 were conducted in the US, 8 studies were from Iran, 6 from India, 4 from Germany, 3 each from UK and South Korea, 2 each from Hong Kong, Italy, Australia, and Brazil, 1 study each conducted in the Netherlands, Portugal, Greece, Spain, Portugal and Spain, France and Canada, and finally three studies that were subsets of the same larger study (98, 116, 117) were conducted simultaneously across multiple countries of the world including Norway, Austria, Australia, Israel, Brazil, Italy, UK, Korea, and USA. Several research groups reported on the exact same sample or on subsets of overlapping samples across multiple papers. Specifically, 4 of the music therapy studies by Srinivasan et al., 2 music studies by Kim et al., 2 yoga-based studies by Radhakrishna et al., 3 martial arts studies by Bahrami et al., and 2 by Phung et al. reported data from the same sample across multiple papers (4, 6, 27, 86, 101, 104, 118–123). Furthermore, 3 music therapy papers (98, 116, 117) reported on samples collected as part of the same, large-scale international study, 3 theater-papers reported on data collected across multiple cohorts by Corbett et al. (124–126) and 2 more martial arts studies had an overlap in reported samples (127, 128).

### Sample Characteristics

The 72 studies had a total sample size of 1,939 participants with ASD. Among the studies that did provide gender-related data (total *N* = 1,573), there were 1,338 males and 235 females. Sixty-six studies were conducted in children between 3 and 21 years, 5 studies included both children and adults, and only 1 study was conducted purely in adults with ASD (see Table 1). Specifically, the ages of participants across CMT approaches were as follows: music (3–38 years), yoga (3–23 years), martial arts (5–17 years), theater (6–21 years), and dance (8–65 years), indicating that within the studies that met our inclusion criteria, music, yoga and dance approaches were the three types of CMT approaches that have been implemented in adults with ASD. All studies provided interventions to individuals with ASD only, except one study that provided training to both individuals with ASD and their caregivers (146). All studies reported that participants did not have prior exposure to CMT.

**Table 1:** Study and sample characteristics.

**References**	**Study location**	**Study design**	**Final sample size (EG, CG)**	**Age [M (SD); range]**	**Diagnosis of subjects**	**Measures used to establish diagnosis**	**Duration in weeks (frequency of sessions/week)**	**Session time in minutes (format—I/G)**	**Intervention type**	**Intervention provider**	**C-group intervention**
**Music/rhythm therapy**											
Edgerton et al. (129)	USA	Reversal	11 (11,0)	6–9	ASD	ND	10 (1)	30 (I)	Nordoff-Robin's IMT	SI	NA
Hartshorn et al. (130)	USA	CCT	76 (38,38)	5; 3–7	ASD	ND	8 (2)	30 (G)	Music/Rhythm-based MT	SI	WLC
Boso et al. (131)	Italy	Pre-post	8 (8,0)	30.2 (5.5); 23–38	ASD	CARS, DSM-IV	52 (1)	60 (G)	Active MT	LC	NA
Kim et al. (119)	South Korea	RCT	10 (5,5)	4.22 (12.1); 3–6	ASD	CARS, ADOS, DSM-IV	12 (1)	30 (I)	IMT	SI	Cross-over design: toy play and MT
Kim et al. (101)											
Gattino et al. (132)	Brazil	RCT	24 (12,12)	9.8 (1.4); 6.8–12.2	AD, AS, PDD-NOS	CARS-BR, ADI-BR, DSM-IV TR	16 (1)	30 (I)	RMT	SI	Routine clinical activities
Hillier et al. (133)	USA	Pre-post	22 (22,0)	18; 13–29	AD, AS, PDD-NOS	DSM-IV	8 (1)	90 (I)	Soundscape- MT program	SI	NA
Wan et al. (134)	USA	Pre-post	6 (6,0)	6.7 (1.2); 5–9	ASD	CARS, DSM-IV	8 (1)	45 (I)	AMMT	LC	NA
Thompson et al. (135)	Australia	RCT	21 (11,10)	3–5	ASD	DSM-IV-TR	16 (1)	30–40 (not clear)	Family centered MT-based movement therapy + Early intervention	SI	Regular EI
LaGasse (136)	USA	RCT	17 (9,8)	7.6 (1.1); 6–9	ASD	CARS	5 (2)	50 (not clear)	MT	SI, O (support staff)	No-music social skills intervention
Ghasemtabar et al. (137)	Iran	CCT	27 (13,14)	7–12	ASD	CARS	6 (2)	30 (G)	MT	SI	NIC
Srinivasan et al. (27)	USA	RCT	36 (12,12,12)	7.7 (2.2); 5–12	ASD	ADOS-2, SCQ	8 (4)	45 (I)	RI	LC, CG, O (model)	Academic sedentary activities, Robot-mediated interactions
Srinivasan et al. (118)											
Srinivasan et al. (4)											
Srinivasan et al. (6)											
Bieleninik et al. (116)	Australia, Austria, Israel, Brazil, Italy Norway, UK, Korea, USA	RCT	314 (165,149)	5.4 (0.9); 4–6	ASD	ADOS, ADI-R, ICD-10	20 (1-low intensity; 3-high intensity)	30 (low); 60 (high); (I)	IMT + standardized care	SI, LC	Enhanced standard care
Mossler et al. (117)	Australia, Austria, Brazil, Israel, Italy, Korea, Norway, UK and USA	Pre-post	101 (101,0)	5.4 (0.3); 4–7	ASD	ADOS, ADI-R, Physician report	20 (1-low intensity; 3-high intensity)	30 (low); 60 (high); (I)	IMT	SI	NA
Dvir et al. (98)	Israel, Austria and Norway	Pre-post	21 (21,0)	5.33 (0.72); 4.1–6.9	ASD	ADOS, ADI-R, Physician report	20 (61,2,3)	30 (I)	MT	SI	NA
Yoo & Kim (138)	South Korea	Pre-post	8 (8,0)	10.8; (3.4)	ASD	KCARS, DSM-IV	8	30 (I)	RI	SI	NA
Willemin et al. (139)	USA	Pre-post	14 (14,0)	10; 5–14	ASD	ND	4 (2)	60 (I)	Drumtastic^®^-drumming program	LC, SI	NA
Lowry et al. (140)	UK	CCT	18 (12,6)	7–8	O	ND	5 (2)	30 (not clear)	Rock drumming	SI	School-based educational program
Stephen (141)	India	CCT	30 (15,15)	ND	ASD	ND	12	ND	ND	ND	WLC
Schmid et al. (142)	USA	Pre-post	64 (64,0)	8.04 (1.62); 5–11	ASD	ASD diagnosis on IEP	16 (1)	45 (G)	Voices together MT	SI	NA
Rabeyron et al. (143)	France	RCT	36 (19,17)	4–7	ASD	CARS	8 (1)	30 (G)	IMT	SI	Music listening
Cibrian et al. (144)	USA	RCT	22 (11,11)	5.72 (1.2)	ASD	DSM-V	8 (1)	(G)	NMT using bendable sound prototype	SI, O (school psychology teachers)	NMT using tambourines
**Yoga/mindfulness-based therapy**											
Radhakrishna et al. (120)	India	CCT	12 (6,6)	8–14	ASD	ICD-10	82 (2)	1 (I)	IAYT + ABA	SI, CG	ABA training
Radhakrishna et al. (121)	India	Pre-post	6 (6,0)	12.7; 8–14	ASD	CARS, DSM-IV TR	40 (5)	45 (not clear)	IAYT	LC, CG	NA
Rosenblatt et al. (145)	USA	Pre-post	24 (24,0)	8.9; 3–16	ASD	70%-physician-provided diagnosis 30%-ND	8 (1)	45 (G)	Relaxation response based-yoga	SI	NA
Koenig et al. (88)	USA	CCT	46 (24,22)	5–12	ASD	ND	16 (5)	15–20 (G)	GRTL Yoga	LC	Standard morning activity at school
de Bruin et al. (146)	Netherlands	Pre-post	21 children with 26 parents (EG: 21	Children−15.8 (2.7); 11–23, Fathers−53.1 (4.4); 48–61,	AD, AS, PDD-NOS, O	AODS-G, DSM-IV TR	9 (1)	90 (G)	*MYmind*—Mindfulness training	LC	NA
			children with 26 parents, CG: 0)	Mothers−49.8 (5.6); 40–56							
Narasingharao et al. (147)	India	CCT	61 (32,29)	5–16	ASD	ICD-10	24 (5)	75 (G)	Yoga	SI, CG	Regular school curriculum
Sotoodeh et al. (148)	Iran	RCT	29 (15,14)	11.2 (2.9); 7–15	ASD	ADI-R, DSM-V	8 (3)	30 (I)	Yoga	SI	NIC
Litchke et al. (149)	USA	Pre-post	5 (5,0)	10.4 (1.8); 8–13	AS, PDD-NOS	ND	4 (2)	1 (G)	Teen yoga warriors—multimodal mandala yoga	LC, O (graduate student)	NA
Kaur & Bhat (150)	USA	RCT	23 (11,12)	5–13	ASD	SCQ, ADOS, Medical Records	8 (4)	40–45 (Expert); (I); 20–25 (Parent)	Creative yoga	LC, O (under-graduate student)	Academic sedentary activities
Vidyashree et al. (151)	India	RCT	35 (15,20)	9.6 (2.4); 8–14	ASD	ND	12	40 (not clear)	Yoga	SI	Routine rehabilitation therapy
Tanksale et al. (152)	Australia	RCT	61 (31,30)	9.42 (1.34); 8–12	ASD	ADOS	6 (1)	60 (G)	Yoga therapy	LC, O (parent, psychology student volunteers)	WLC
**Martial art**											
Bahrami et al. (122)	Iran	RCT	30 (15,15)	9.1 (3.3); 5–1	ASD	GARS, DSM-IV TR	14 (4)	30 (I)	Heian Shodan Kata technique	SI	Educational intervention
Movahedi et al. (153)			26 (13,13)	9.03 (3.3); 5–16				30, 90 (I, G)			
Bahrami et al. (86)			30 (15,15)	9.1 (3.3); 5–16				30, 90 (I, G)			
Chan et al. (154)	Hong Kong	RCT	40 (20,20)	6–17	AD, PDD-NOS	ADI-R, DSM-IV TR	4 (2)	60 (G)	Nei Yang Gong- Mind-body exercise	LC	PMR
Chan et al. (155)	Hong Kong	RCT	48 (18,17,13)	5–17	AD, PDD-NOS	ADI-R, DSM-IV TR	4 (2)	60 (G)	Chanwuyi- Mind-body exercise	LC	PMR, NIC
Figueiredo et al. (156)	Portugal and Spain	Pre-post	8 (8,0)	8.5 (1.6); 7–12	ASD, AS, O	ND	6–18 (1)	30–45 (not clear)	Karate	ND	NA
Kim et al. (157)	USA	CCT	14 (8,6)	8–14	ASD	Physician report	8 (2)	50 (not clear)	Taekwondo	SI	NIC
Phung & Goldberg (104)	USA	RCT	34 (14,20)	9.3 (1.1); 8–11	ASD	SCQ, ADOS-2, Clinician Report	13 (2)	45 (G)	Mixed martial arts	SI, O (peers, under-graduate students)	WLC
Phung et al. (123)											
Sarabzadeh et al. (158)	Iran	RCT	18 (9,9)	6–12	ASD	GARS, Physician Report	6 (3)	60 (not clear)	Tai Chi Chuan	SI	NIC
Garcia et al. (127)	USA	Pre-post	14 (14,0)	12.3 (3.4); 8–17	ASD, O	Physician report	8 (1)	45 (G)	Judo	SI, O (graduate student)	NA
Rivera et al. (128)	USA	Pre-post	33 (33,0)	12;67 (2.99); 8–17	ASD	Physician report	8 (1)	45	Judo	SI	NA
Ansari et al. (159)	Iran	RCT	30 (15,15)	8–14	ASD	Physician report	10 (2)	60	Kata technique	SI	Aquatic therapy, WLC
AdibSaber et al. (160)	Iran	RCT	20 (10,10)	8–14	ASD	GARS-2	10 (2)	60 (G)	Heian Shodan Kata technique	ND	Maintained regular program and activity levels
Greco & de Ronzi (161)	Italy	RCT	28 (14,14)	9.25 (1); 8–11	ASD	ADOS-2	12 (2)	45 (G)	Karate	SI	WLC
Tabeshian et al. (162)	Canada	RCT	23 (12,11)	9.6 (1.4); 6–12	ASD	Physician report	12 (3)	45 (G)	Tai Chi Chuan	SI	WLC
**Theater/dramatic training**											
Lerner et al. (163)	USA	CCT	17 (9,8)	11–17	AD, AS	DSM-IV TR	6 (5)	300 (G)	SDARI	SI	NIC
Lerner & Mikami (164)	USA	RCT	13 (7,6)	ND	AD, AS, PDD-NOS	SCQ, LC	4 (1)	90 (G)		SI	Skill-streaming
Corbett et al. (124)	USA	Pre-post	8 (8,0)	11.3 (4); 6–17	AD, PDD-NOS	ADOS-G, DSM-IV TR	12 (1-4)	120 (I)	SENSE Theater	CG, O (peers)	NA
Corbett et al. (126)	USA	Pre-post	11 (11,0)	12.1; (8–17)	AD, AS, PDD-NOS	ADOS-G, DSM-IV	2 (5)	240 (I, G)		LC, O (peers)	NA
Corbett et al. (165)	USA	RCT	30 (17,13)	8–14	ASD	ADOS, DSM-V	10 (1)	240 (I, G)		T, O (peers)	NIC
Corbett et al. (87)											
Ioannou et al. (125)	UK	RCT	77 (44,33)	8–16	ASD	ADOS-2, physician report	10 days	240 (G)	SENSE Theater	O (peers)	WLC
Guli et al. (166)	USA	CCT	34 (18,16)	10.9; 8–14	ASD, O	DSM-IV TR	Fall: 8 (2) Spring: 12 (1)	Fall: 90 Spring: 120 (G)	SCIP	SI	WLC
Kim et al. (167)	USA	Pre-post	18 (18,0)	15	ASD	ND	5 (5)	4 (G)	Theater	SI	NA
Reading et al. (168)	USA	CCT	16 (8,8)	17–21	ASD	ND	10 (1)	2 (G)	Theater	SI	NIC
Naniwadekar et al. (169)	India	CCT	8 (ND)	ND	ASD	ND	ND	ND (G)	Drama	SI	Story telling using flash cards and video
Beadle-Brown et al. (170)	UK	Pre-post	22 (22,0)	7–13	ASD	ADOS	10 (1)	45 (I)	“Imagining Autism” —Drama	SI	NA
**Dance therapy**											
Arzoglou et al. (85)	Greece	CCT	10 (5,5)	16.8 (EG), 16.6 (CG)	ASD	DSM-IV	8 (3)	35–45 (I, G)	Greek traditional dance	ND	Physical education at school
Koehne et al. (171)	Germany	CCT	51 (27,24)	18–55	AD, AS	ADOS, ADI-R, DSM-IV/ICD-10	10 (1)	90 (G)	Synchrony-based DMT	ND	Movement intervention without imitation or synchronization
Koch et al. (77)	Germany	CCT	31 (16,15)	22 (7.7); 16–47	ASD, AD, AS	ICD-10	7 (1)	60 (G)	M-DMT	SI	WLC
Hildebrandt et al. (172)	Germany	RCT	43 (31,12)	22.5 (7.75); 14–65	ASD	ICD-10	10 (1)	60 (G)		SI	WLC
Mastrominico et al. (173)	Germany	RCT	56 (35,21)	22.5 (8.5); 14–52	ASD	ICD-10, SANS	10 (1)	60 (G)		SI	WLC
Souza-Santos et al. (174)	Brazil	Cross-over	45 (15,15,15)	7 (1.1)	ASD	CARS, DSM-V	12 (2)	60 (not clear)	Dance + EAT	SI	CG1: EAT, CG2: EAT and Dance
Aithal et al. (175)	UK	Cross-over	26 (10,16)	10.65; 8–13	ASD	DSM-V	5 (2)	40 (G)	Dance movement psychotherapy	SI	Standard care
**Miscellaneous: dance and music therapy**											
Mateos-Moreno & Atencia-Doña (176)	Spain	CCT	16 (8,8)	ND	ASD	CARS, DSM-IV	17 (2)	60 (G)	Dance + Music (combined)	SI, O (graduate student)	NIC

*ABA, Applied Behavioral Analysis; AD, Autistic Disorder; ADI-R, Autism Diagnostic Interview-Review; ADI-BR, Brazilian version of ADI-R; ADOS, Autism Diagnostic Observation Schedule; AMMT, Auditory-motor Mapping Training; ASD, Autism Spectrum Disorder; AS, Asperger's Syndrome; CARS, Childhood Autism Rating Scale; CARS-BR, Brazilian version of CARS; KCARS, Korean version of CARS; CCT, Controlled Clinical Trial; CG, Control Group; Cg, Caregiver; DMT, Dance Movement Therapy; DSM, Diagnostic and Statistical Manual of Mental Disorders; EAT, Equine-assisted therapy, EG, Experimental group; ESDM, Early Start Denver Model; G, Group therapy; GARS, Gilliam Autism Rating Scale; GRTL, Get Ready To Learn; I, Individual therapy; IAYT, Integrated approach to Yoga Therapy; ICD, International Classification of Diseases; IEP, Individualized Education Program; IMT, Improvisational Music Therapy; LC, Licensed Clinician; M-DMT, Manualized Dance Movement Therapy; MT, Music Therapy; NA, Not Applicable; ND, Not Defined; NMT, Neurologic Music Therapy; NS, Not specified; NIC, No Intervention Control; O, Others; PDD, Pervasive Developmental Disorder; PDD-NOS, Pervasive Developmental Disorder-Not Otherwise Specified; PMR, Progressive muscle relaxation; RCT, Randomized Controlled Trial; RI, Rhythm Intervention; RMT, Relational Music Therapy; SANS, Scale for Assessment of Negative Symptoms; SCIP, Social Competence Intervention Program SCQ, Social Communication Questionnaire; SDARI, Socio-dramatic Affective Relational Intervention; SENSE, Social Emotional NeuroScience Endocrinology; SI, Specialized Instructor; T, Teacher; WLC, Waitlist Control*.

Sixty-seven studies recruited only individuals with ASD and the remaining 5 studies included children with ASD as well as children with other diagnoses including ADHD, anxiety disorder, learning disability, sensory processing disorder, and emotional and behavioral disorder. Across studies, the diagnosis of ASD was confirmed using multiple measures including standardized tests, physician report and parent-report questionnaires (see Table 1). Specifically, 41 studies employed gold standard measures such as the Autism Diagnostic Observation Schedule (ADOS), Autism Diagnostic Interview-Revised (ADI-R), Gilliam Autism Rating Scale (GARS), and Childhood Autism Rating Scale (CARS) to confirm ASD diagnosis, 19 studies relied on physician diagnosis made using criteria listed in the Diagnostic and Statistical Manual of Mental Disorders (DSM) or the International Classification of Disease (ICD), and 12 studies did not provide details of methods used to confirm participants' ASD diagnosis (see Table 1). In terms of intellectual functioning of participants, only 27 of the 72 studies reported on assessing Intellectual Quotient (IQ) scores using various scales such as Wechsler Abbreviated Scale of Intelligence-2nd edition (WASI-2) or the PsychoEducational Profile (PEP). Overall, only five studies included children with mild intellectual disability in their sample, with the remaining studies including participants without any accompanying intellectual disability (101, 117, 119, 150, 170). Although a vast majority of included studies did not report on socioeconomic status, the remaining studies primarily included participants from middle and upper-middle class families.

Study sample sizes across the different CMT interventions were as follows: 764 participants in music therapy interventions (455 received experimental intervention and 309 received control interventions), 317 in yoga therapy (184 in experimental group and 133 controls), 326 in martial arts (176 in experimental group and 150 control group participants), 246 participants in theater training (162 received experimental intervention and 84 were in control group; 1 theater study (*N* = 8) did not provide the distribution of the sample into the intervention groups), 262 participated in dance-based studies (139 received experimental group intervention and 123 received a control intervention), and 16 participated in combined music and dance intervention (8 in experimental and 8 in control group). There was great variability in sample sizes across individual studies. The largest sample size studies for different CMT approaches included 364 participants for music therapies (116), 61 participants for yoga therapy (147, 152), 57 participants for dance therapy (173), 48 participants for martial arts (155), 77 participants for theater–based interventions (125), and 56 participants for dance and other combined therapies (173).

### Study Characteristics

Out of the 72 studies, 34 studies were RCTs, 17 were CCTs, 18 were pre-post designs, 2 studies were cross-over designs, and 1 study employed a reversal design. We scored the PEDro scale for the 52 clinical trials (16 music, 7 yoga, 13 martial arts, 8 theater, 7 dance, and 1 combined dance and music intervention) and the NIH quality assessment tool for the 20 single group pre-post design studies (9 music, 4 yoga, 3 martial arts, 4 theater) reviewed to assess risk of bias (see Tables 2A,B). The clinical trials included in the review employed the following control groups: waitlist control, ABA therapy, routine or enhanced standard-of-care, seated play, school-based educational programming, social skills training, physical education training, robotic therapy, equine-assisted therapy, or no intervention. In terms of fidelity of implementation of training procedures, of the total 72 studies, around 25% (*N* = 18) used and provided details of specific checklists employed to monitor the consistency of treatment implementation, another 22% (*N* = 16) provided brief details of some form of fidelity checks, and 51% (*N* = 38) of studies did not provide any information on intervention fidelity. In terms of intervention implementation, music therapy and yoga therapy-based studies were almost equally split between an individualized vs. group-based format (Music: 13 out of 25 studies and yoga: 5 out of 11 studies provided individualized intervention), whereas martial arts (12 out of 16 studies), theater (10 out of 12 studies), dance (6 out of 7 studies), and combination-based approaches frequently employed group-based implementation with group sizes varying between 3 and 12 participants (see Table 1).

**TABLE 2A:** PEDro scoring for RCT/CCT.

**References**	Eligibility criteria specified^**^	**Random subject allocation**	**Concealed allocation**	**Baseline similarity of groups**	**Blinding: subjects**	**Blinding: therapists**	**Blinding: assessors**	**Measures of key outcomes**	**Intent to treat**	**Between group analyses**	**Point estimates and variability measures**	**Total (ROB)**
**Music**												
Hartshorn et al. (130)												4 (H)
Kim et al. (119)												6 (L)
Kim et al. (101)												4 (H)
Gattino et al. (132)												8 (L)
Thompson et al. (135)												7 (L)
LaGasse (136)												6 (L)
Ghasemtabar et al. (137)												5 (H)
Srinivasan et al. (27)												7 (L)
Srinivasan et al. (118)												7 (L)
Srinivasan et al. (4)												7 (L)
Srinivasan et al. (6)												7 (L)
Bieleninik et al. (116)												8 (L)
Stephen (141)												4 (H)
Lowry et al. (140)												5 (H)
Rabeyron et al. (143)												5 (H)
Cibrian et al. (144)												4 (H)
**Yoga/mindfulness-based therapy**												
Radhakrishna et al. (120)												1 (H)
Koenig et al. (88)												4 (H)
Narasingharao et al. (147)												2 (H)
Sotoodeh et al. (148)												6 (L)
Kaur & Bhat (150)												6 (L)
Vidyashree et al. (151)												4 (H)
Tanksale et al. (152)												6 (L)
**Martial arts**												
Bahrami et al. (122)												5 (H)
Movahedi et al. (153)												5 (H)
Bahrami et al. (86)												5 (H)
Chan et al. (154)												8 (L)
Chan et al. (155)												6 (L)
Kim et al. (157)												4 (H)
Phung & Goldberg (104)												6 (L)
Phung et al. (123)												6 (L)
Sarabzadeh et al. (158)												8 (L)
Ansari et al. (159)												6 (L)
AdibSaber et al. (160)												5 (H)
Greco & de Ronzi (161)												9 (L)
Tabeshian et al. (162)												8 (L)
**Theater/dramatic training**												
Lerner et al. (163)												6 (L)
Lerner & Mikami (164)												7 (L)
Guli et al. (166)												8 (L)
Corbett et al. (165)												7 (L)
Corbett et al. (87)												4 (H)
Ioannou et al. (125)												5 (H)
Reading et al. (168)												5 (H)
Naniwadekar et al. (169)												4 (H)
**Dance therapy**												
Arzoglou et al. (85)												4 (H)
Koehne et al. (171)												7 (L)
Koch et al. (77)												5 (H)
Hildebrandt et al. (172)												5 (H)
Mastrominico et al. (173)												5 (H)
Souza-Santos et al. (174)												5 (H)
Aithal et al. (175)												7 (L)
**Miscellaneous interventions**												
Mateos-Moreno & Atencia-Doña (176)												4 (H)

** *Item 1 is not included in PEDro total score calculation*.

*Gray shaded cells indicate that the criterion has been fully satisfied (receives a score of 1 point for that item) and blank cells indicate that the criterion has not been satisfied (receives a score of 0 points for that item). Total scores are calculated by summing the number of gray shaded cells (except criterion 1) for each individual study*.

*ROB, Risk of bias, H, High risk of bias, L, Low risk of bias. ROB rating based on PEDro scores (i.e., Low risk = scores ≥ 6, High risk = scores < 6)*.

**TABLE 2B:** NIH quality assessment tool for before-after (pre-post) study design.

**References**	**Study objective**	**Eligibility criteria**	**Sample represen-tation**	**Eligible partici-pants enrolled**	**Sample size/power analysis**	**Intervention description**	**Outcome measures specified**	**Blinding: assessors**	**Intent to treat**	**Statistical tests used**	**Multiple assessments of outcome measures**	**Group/ individual level analysis**	**Total and ROB**
**Music therapy**												
Edgerton et al. (129)													8 (M)
Boso et al. (131)													8 (M)
Hillier et al. (133)													8.5 (M)
Wan et al. (134)													8 (M)
Yoo and Kim (138)													8.5 (M)
Willemin et al. (139)													8 (M)
Schmid et al. (142)													9.5 (L)
Dvir et al. (98)													9.5 (L)
Mossler et al. (117)													11 (L)
**Yoga therapy**												
Radhakrishna et al. (121)													7 (M)
Rosenblatt et al. (145)													9 (L)
de Bruin et al. (146)													9 (L)
Litchke et al. (149)													8 (M)
**Martial arts**												
Figueiredo et al. (156)													6 (H)
Garcia et al. (127)													9 (L)
Rivera et al. (128)													8 (M)
**Theater**												
Corbett et al. (124)													8 (M)
Corbett et al. (126)													8 (M)
Kim et al. (167)													7.5 (M)
Beadle-Brown et al. (170)													10.5 (L)

*Gray shaded cells indicate that the criterion has been fully satisfied (receives a score of 1 point for that item), cells with diagonal stripes indicate that the criterion was partially satisfied (receives a score of 0.5) and blank cells indicate that the criterion has not been satisfied (receives a score of 0 points for that item). Total scores are calculated by summing the number of gray shaded cells (except criterion 1) for each individual study*.

*ROB, Risk of bias; H, High risk of bias; M, Moderate risk of bias; L, Low risk of bias. Risk of bias rating based on scoring on ratings on NIH quality assessment tool for pre-post designs (i.e., Low risk = scores ≥ 9, High risk = scores ≤ 6, Moderate risk = scores of 6.1–8.9)*.

### Risk of Bias

#### Controlled Intervention Studies

Out of the 52 clinical trials, 50% studies (*N* = 26) had a high risk of bias (PEDro scores <6). No study satisfied all the 11 criteria (see Table 2A). Among factors contributing to risk of bias, few studies concealed allocation of subjects to intervention groups (*N* = 8), and blinding of subjects (*N* = 1), therapists (*N* = 9), and assessors (*N* = 20) were ensured to a varying extent by reviewed studies. Although not as frequent, other factors associated with risk of bias included random subject allocation (*N* = 36 satisfied the criterion) and baseline similarity of groups on key prognostic measures (*N* = 33 satisfied the criterion) (see Table 2A).

#### Single Group Pre-post Designs

Based on the NIH quality assessment tool rating used for assessing the 20 pre-post designs, 1 study was rated as “poor” indicating high risk of bias, 12 studies were “fair” indicating moderate risk of bias, and 7 studies were rated as “good” indicating low risk of bias. Specifically, none of the studies measured outcomes multiple times at pretest and posttest to get stable estimates of child performance, and all but three studies did not discuss power analyses to justify the choice of sample sizes. Another area of concern included blinding of assessors which was ensured in only 3 studies. Finally, 50% studies (*N* = 10) did not report on validity and reliability of assessed outcome measures, with 30% of the remaining studies (*N* = 6) reporting on only one but not both these measures (see Table 2B).

### Intervention Characteristics

The mean duration of studies that provided music-based interventions was 12 weeks (SD = 9.73, Range = 4–52 weeks), with a mean frequency of around 2 sessions/week (SD = 1.3, Range = 2–5 times), and each session lasting around 40 min (SD = 14.9, Range = 30–90 min, see Table 1 for details). Studies that provided yoga therapy had the longest mean intervention duration of 20 weeks (SD = 23.99, Range = 4–82 weeks) with a mean frequency of around 3 times per week (SD = 1.81, Range = 1–5 times) for around 50 min per session (SD = 19.9, Range = 20–90 min). Martial arts and theater-based studies had similar intervention characteristics, i.e., average intervention duration ranged around 10 and 8 weeks respectively (Martial arts: SD = 3.44, Range = 4–14 weeks; Theater: SD = 3.38, Range = 4–12 weeks) and average frequency was around 2 sessions/week (Martial arts: SD = 1.01, Range = 1–4 times; Theater: SD = 1.86, Range = 1–5 times). However, the 2 CMT types differed greatly in terms of average session duration, with theater interventions (Mean ~175 min/session, SD = 90.56, Range = 60–300 min) lasting on an average for much longer time compared to martial arts interventions (Mean ~50 min/session, SD = 9.2, Range = 30–60 min). Lastly, interventions focusing on dance therapy had an overall mean duration of 9.5 weeks (SD = 1.76, Range = 7–12 weeks), with a mean frequency of around 1–2 sessions per week (SD = 0.78, Range = 7–12 weeks), and each session lasting for around 60 min (SD = 16.02, Range = 40–90 min).

In terms of intervention providers, most of the CMT approaches were provided by either licensed clinicians or specialized instructors trained in the CMT approach (*N* = 62). Fifteen studies (6 music, 3 yoga, 2 martial arts, 3 theater, 1 music and dance combined) asked teachers, caregivers, support staff, models, students or peers, etc. to assist in the intervention delivery process (see Table 1). Only 2 theater studies by Corbett et al. had teaching staff and peers deliver the intervention independently of clinicians after conducting a 2-day intensive training seminar (87, 165). Several papers mentioned using conventional ASD treatment strategies while providing CMT interventions to children with ASD. Common training strategies were based on principles of conventional ASD treatments such as ABA, TEACHH, and PECS and specifically included the use of picture schedules/visual cues, incremental prompting (verbal, gestural, modeling, hand-on-hand assistance), reinforcement schedules, structured and predictable training routines, motivational strategies, activities designed keeping in mind the participant's sensory needs, and the use of non-competitive, goal-directed, and child-led activities to ensure child compliance. Although a total of 15 studies (2 music, 2 yoga, 5 martial arts, 4 theater, 1 dance, 1 music and dance) mentioned progression in training across intervention weeks, only four of these studies (1 music, 1 yoga, 1 martial art, 1 theater) discussed specific principles of treatment progression over the course of the program. The remaining 57 studies provided no information on treatment principles and progression.

Common music therapy training approaches evaluated included Improvisational Music Therapy (IMT) and Relational Music Therapy (RMT). Similarly, yoga-based training approaches included Mandala Yoga and Mindfulness training, Relaxation Response-based training, and ABA based- integrated Yoga training. Common martial art approaches included Kata, Judo, Karate, Tai chi, and Taekwondo. Theater-based studies used programs such as the Social Emotional NeuroScience Endocrinology (SENSE) Theater, Social Competence Intervention Program (SCIP), and Socio-dramatic Affective Relational Intervention (SDARI). Lastly, Dance Movement Therapy (DMT) and traditional Greek dance were some of the approaches used in dance-based studies (see Table 1).

In terms of the location of intervention delivery, 5 studies (all music) delivered interventions at the child's home, 13 studies (4 music, 4 yoga, 2 martial arts, 1 theater, 1 dance, 1 music and dance) delivered interventions at the child's school, 30 studies (10 music, 1 yoga, 8 martial arts, 7 theater, 4 dance) provided intervention at other indoor settings such as a community center, YMCA, etc., 3 studies (1 music, 1 martial art, 1 theater) provided intervention either at the child's school or a community center, 2 studies (both yoga-based involving the same sample) conducted their intervention in a calm and open outdoor setting, and 19 of the remaining studies (5 music, 4 yoga, 5 martial arts, 3 theater, 2 dance) did not provide any specific information on where the intervention was provided.

### Outcomes Measures and Treatment Effects

Of the 72 studies reviewed, 31 reported within-group changes, 31 reported between-group differences, and 10 studies reported both between- and within-group changes. Studies used a combination of tests and measures including standardized tests, self-/parent-/teacher-reported questionnaires, video coding, and observational measures to assess the impact of CMT on multiple domains including social communication, behavioral-affective, sensorimotor, cognitive, functional skills, and quality of life (see Tables 3A–C, **6**). Twenty-eight studies (16 music, 3 yoga, 1 martial arts, 6 theater, 1 dance, 1 combined music and dance) reported on the inter- and intra-reliability of the assessments employed. In terms of reporting treatment effects, 28 studies reported ES for the assessed outcomes (see Tables 4A–C). We were able to use data from the original papers to calculate ES in 64 out of the total 72 studies (i.e., 89% studies). Tables 4A–C provides a comparison between ES we calculated based on data provided in the paper and ES estimates reported in the original paper, and also discusses the level of agreement between both sets of estimates. **Table 6** displays our results to indicate the number of studies stratified by CMT approach where calculated ES were statistically significant (i.e., CI did not include 0). Below, we provide a narrative description of the types of measures employed and summarize the salient treatment effects from the studies included in our review across the developmental domains. Please note that each section discusses the results reported in the original papers assessing those domains followed by a summary of the results from our own ES calculations for the specific domain.

**TABLE 3:** Study-wise dependent variables and results.

**References**	**Domains tested**	**Study design**	**Type of effect reported**	**Type of assessment**	**Measures**	**Measures/variables showing improvement**
**(A) Music/rhythm therapy**						
Edgerton et al. (129)	Other (musical and non-musical communication abilities)	W	W	Questionnaire	CRASS	Total CRASS scores
Hartshorn et al. (130)	Social, behavioral, and sensory	B	B	Video coding	Stereotypies, compliance, on-task behavior, eye contact, response to teacher	Wandering, negative response to touch, resisting teacher, on-task passive behavior
Boso et al. (131)	Behavioral and other (severity of illness and music skills)	W	W	Standardized scale, questionnaire	CGI-S, CGI-I, BPRS, musical skills	BPRS and music skills (except complex rhythmic patterns) from PRE to POST
Kim et al. (119)	Social communication and behavioral	B	B	Standardized scale, video coding	PDDBI-C, ESCS, Video coding (eye contact and turn taking)	PDDBI-C, ESCS (RJA, IJA), eye contact and turn taking during sessions
Kim et al. (101)	Social and behavioral	B	B	Video coding	Episodes of joy, compliance, and initiation of engagement	All variables
Gattino et al. (132)	Social communication	B	B	Standardized scale	CARS-BR	Nonverbal communication scores on CARS-BR in EG.
Hillier et al. (133)	Social and behavioral	W	W	Questionnaire	IPR (participant and parent), RSES, STAI-C	All variables
Wan et al. (134)	Communication	W	W	Video coding	Video coding of child's vocal output	All variables
Thompson et al. (135)	Social communication	B	B	Video coding, questionnaire	VSEEC, SRS, Mac-CDI, PCRI, MTDA	VSEEC
			W			MTDA
LaGasse (136)	Social communication	B	B	Video coding, questionnaire	SRS, ATEC, and video coding (eye gaze, JA, communication and withdrawal behaviors)	ATEC (parent and teacher- main effect), eye gaze and joint attention toward persons
			W			SRS
Ghasemtabar et al. (137)	Social	B	B	Questionnaire	SSRS	SSRS—social skills and functioning
Srinivasan et al. (27)	Behavioral	B	B	Video coding, questionnaire	RBS-R, video coding (positive, negative and greater interested affect)	Positive affect (mid and late)
			W			EG: RBS-R (lower negative behaviors from early to mid and late sessions, lower negative and greater interested affect from early to late sessions)
Srinivasan et al. (118)	Motor	B	W	Standardized scale, video coding	BOT-2, video coding (imitation and interpersonal synchrony)	EG: BCC, imitation, IPS CG: FMCC, imitation
Srinivasan et al. (4)	Social	B	B	Standardized scale, video coding	JTAT, video coding (spontaneous and responsive social attention)	Attention to targets: EG—greater attention to social partners and CG—greater attention to objects in the early, middle, and late sessions, Increased Spontaneous and Responsive Attention (EG)
			W			JTAT (EG)
Srinivasan et al. (6)	Social communication	B	W	Standardized scale, video coding	JTAT, video coding (response to social bids, verbalization and vocalization)	Increase in response to social bids from early to mid and late sessions, respectively, in EG and CG; socially-directed verbalization increased in EG, self-directed verbalization greater in CG, JTAT in EG
Bieleninik et al. (116)	Social, QOL, other (cost-effectiveness and parent reported adverse events)	B	B	Standardized scale, questionnaire	ADOS, SRS (parent), QOL, cost-effectiveness scores	QOL (5M), ADOS—social affect (5M), SRS—motivation (5M) (as) and mannerisms (Baseline to 2M, 5M, and 12M) (as)
Mossler et al. (117)	Social communication	W	W	Standardized scale, questionnaire	ADOS, AQR, SRS	Attunement with therapist associated with changes in SRS total and cognition sub-scale scores at 5M (as)
Dvir et al. (98)	Social communication and motor	W	W	Standardized scale, video coding	ADOS-2, movement analysis, attunement analysis	Attunement parameters: AI and TAI, ADOS-CS (5M)
Yoo & Kim (138)	Social and motor	W	W	Observational assessment, video coding, questionnaire	K-SSRS, imitation, drumming, video coding (eye gaze and joint action synchronous movement), social validity	K-SSRS (total scores, self-control and cooperation subscales)
Willemin et al. (139)	Social and behavioral	W	W	Questionnaire	SPRS, fun-o-meter, smiley-o-meter, PANAS-C	SPRS, smiley-o-meter, fun-o-meter
Lowry et al. (140)	Social, motor, and other (program effectiveness and feasibility)	B	B	Standardized scale, video coding, questionnaire	MABC-2, drumming skills, SDQ, staff interview	Drumming (peer drum and EBD control at posttest and FUP) and SDQ (EBD drum- total difficulties and hyperactivity)
Stephen (141)	Social communication	B	B	Questionnaire	SSRS	EG: social skills
Schmid et al. (142)	Social communication	W	W	Video coding, questionnaire	DUACS, PDDBI, spoken language questionnaire	Language levels, empathy, social pragmatic problems, social awareness problems
Rabeyron et al. (143)	Social communication, behavioral	B	B	Standardized scale, questionnaire	CGI, ABC, CARS	CGI, CARS total, ABC total, lethargy, stereotypy
Cibrian et al. (144)	Motor and others (engagement with music)	B	B	Questionnaire, video coding	Bendable sound survey, DCDQ, strength, reaction time	DCDQ scores, control of movement, fine motor skills, strength, reaction time
**(B) Yoga/mindfulness and martial arts-based interventions**						
**Yoga/mindfulness-based therapy**						
Radhakrishna et al. (120)	Social communication, behavioral and motor	B	W	Observational assessment, questionnaire	ARI-E2 checklist, ITB, RSB	Qualitative improvements noted on all variables but details per outcome measure not provided
Radhakrishna et al. (121)	Motor	W	W	Observational assessment, questionnaire	ITB, parent-reported improvements using custom rating scale	ITB—improved imitation skills in GM, oro-facial, breathing, complex motor, and vocalization domains. Qualitative improvements in JA, object use, play, compliance, and language
Rosenblatt et al. (145)	Behavioral	W	W	Questionnaire/interview	BASC-2, ABC	BASC-2 (total sample—BSI; latency group—BSI, internalizing), ABC (latency group—irritability) (as)
Koenig et al. (88)	Behavioral	B	B	Video coding, questionnaire	ABC-C (parent and teacher), video coding (off-task behaviors and Teacher redirection)	ABC-C (teacher- total, irritability/agitation/crying, lethargy/social withdrawal and hyperactivity/non-compliance), Off-task behaviors, Teacher redirection
de Bruin et al. (146)	Social, behavioral, QOL, and others (self-reported anxiety and worry, mindful awareness, parental stress)	W	W	Questionnaire	Children: AQ, MAAS, PSWQ, RRS, WHO-5, parents rating children: SRS, AQ, parents rating themselves: FFMQ, WHO-5, IM-P, PSI-C, parenting scale	Children: WH0-5, RRS, parent rating children: SRS, parents rating themselves: FFMQ, WHO-5 (as)
Narasingharao et al. (147)	Behavioral and others (GI and sleep problems)	B	W	Questionnaire	Custom questionnaire	Custom questionnaire (sleep, food, digestion, behavior (except savant ability)
Sotoodeh et al. (148)	Social communication, motor, and cognitive	B	B	Questionnaire	ATEC	ATEC (total scores, sociability, sensory/cognitive awareness, and healthy/physical behavior subscales)
Litchke et al. (149)	Social and behavioral	W	W	Video coding, questionnaire	TSSA, MFMS	TSSA (total, response to initiation, initiating interaction, affective understanding and perspective taking), MFMS (positive mood) (as)
Kaur & Bhat (150)	Motor	B	W	Standardized scale, video coding	BOT-2, video coding (% imitation error)	BOT-2 (EG: BC subtest, CG: FMI and FMP subtests), % imitation error EG: early-mid and early-late, CG: early-late, CG: showed positive correlation between IQ levels and BOT-2 FMI scores, % imitation error CG: mid-late (as)
Vidyashree et al. (151)	Others (HR variability)	B	W	ECG	ECG recording in Lead II	ECG (EG): Reduction in HR and pNN50%, Increase in mean RR, SDNN, RMSSD.
Tanksale et al. (152)	Cognitive and others (sleeping quality, anxiety, goal attainment scale, and emotion awareness)	B	B	Questionnaires	BRIEF-2, children's sleep habits questionnaire, anxiety scale for children with ASD-parent and self-report, GAS, emotional awareness questionnaire	BRIEF-2 (GEC and organization of material subscale at posttest and FUP, the self-monitor, working memory and task monitor subscale scores at posttest), children's Sleep Habits questionnaire (bedtime resistance, sleep onset delay, sleep breathing disorder), emotion awareness (verbal sharing and willingness to understand emotions), anxiety scale
**Martial arts**						
Bahrami et al. (122)	Behavioral	B	W	Standardized scale	GARS (stereotypy)	GARS (stereotypy- pre to post and FUP)
Movahedi et al. (153)	Social	B	W	Standardized scale	GARS (social interaction)	GARS (social interaction—pre to post and FUP)
Bahrami et al. (86)	Communication	B	W	Standardized scale	GARS (communication)	GARS (communication—pre to post and FUP)
Chan et al. (154)	Social communication, behavioral, cognitive	B	B	Computerized test, questionnaires, observational assessments, and EEG	Neuropsychological measures, ATEC, custom questionnaire, Go-No-Go test, EEG	Neuropsychological measures (TOL- rule violation), custom questionnaire (temper outburst), Neuropsychological measures [TOL-initial time (as)]
			W			ACC (EG: No-Go part of Go-No-Go Test), Main Effect of time- Neuropsychological measures (EG: CCTT-T2, FPT), ATEC (EG: sensory/cognitive awareness, sociability, health/physical behavior subscales) [TOL-initial time (as)]
Chan et al. (155)	Cognitive	B	W	Computerized test, EEG	Memory functions and EEG measures	Memory functions (visual scanning and semantic clustering), EEG measures (Theta coherence in left fronto-posterior region, left to right fronto-posterior region, frontal and posterior scalp). Increased theta source activity in BL prefrontal cortex, left parietal cortex and medial and inferior temporal cortex in EG. In CG, increased source activity in left medial and inferior temporal cortex
Figueiredo et al. (156)	Social and behavioral	W	W	Questionnaire	Conner's scale (parent), SDQ	Conner's scale—parent (oppositional, DSM-IV total and defiant/aggressive behaviors), SDQ (peer relationship)
Kim et al. (157)	Motor	B	B	Posturography	Static balance test, functional balance test	Static balance test (single leg—R (eyes closed)
			W			EG: static balance test [single leg—L (eyes open), double leg—unstable surface (eyes closed) (as)], Functional balance test (step-quick turn to R (as)
Phung & Goldberg (104)	Cognitive	B	B	Questionnaire, computerized tests	Hearts and flowers executive functioning test, BRIEF-2	% accuracy scores on congruent and mixed blocks of the Hearts and flowers test, BRIEF-2 (global executive, behavior, and emotion regulation index)
Phung et al. (123)	Social communication	B	B	Questionnaire	SSIS	EG: SSIS
Sarabzadeh et al. (158)	Motor	B	B	Standardized scale	MABC-2	MABC-2 (total, ball skills, and balance)
			W			EG: MABC-2 (total, ball skills, and balance)
Garcia et al. (127)	Motor and others (continued participation in a similar program)	W	W	Physical activity monitoring using actigraph	MVPA	Increase in % time spent and increase in number of minutes (as) spent in MVPA/day
Rivera et al. (128)	Social communication	W	W	Questionnaire	ABC, Parent perspective questionnaire	–
Ansari et al. (159)	Motor	B	B	Qualitative measures	Static and Dynamic balance	EG: static and dynamic balance with greater improvements in kata group than aquatic training group compared to the control group
AdibSaber et al. (160)	Others (sleep habits)	B	B	Questionnaire	Sleep habits questionnaire	EG: sleep resistance, sleep duration, sleep anxiety, night time awakening, parasomnia, and daytime sleepiness
Greco & de Ronzi (161)	Social communication and cognitive	B	B	Questionnaire	SSIS-RS, BRIEF	EG: SSIS-RS (Social skills, problem behaviors scale), BRIEF (behavior regulation index, emotion regulation index, cognitive regulation index, global executive functioning composite)
Tabeshian et al. (162)	Behavior	B	B	Standardized scale	GARS-2	EG: GARS-2 stereotypy (pre-post)
			W			
**(C) Theater/dramatic, dance-based, and miscellaneous interventions**						
**Theater/dramatic training**						
Lerner et al. (163)	Social communication and behavioral	B	B	Questionnaire, computerized tests	EDI, SRS, SSRS, BDI-Y, satisfaction survey, CBCL, DANVA-2	Time 1–7: all measures, time 1–5: EDI (non-verbal communication), CBCL (Internalizing), SSRS (overall), DANVA-2 (child faces, postures)
Lerner & Mikami (164)	Social	B	B	Video coding, questionnaire	SRS, SSRS (parent and teacher), SIOS, Socio-metrics (child's social and friendship preferences)	SIOS: positive and negative interactions, socio-metrics: social preference (as), reciprocal friendship nominations. SSRS-T (social skills)
Corbett et al. (124)	Social, behavioral, sensory problems and other (stress levels)	W	W	Standardized scale, questionnaire	NEPSY, SRS, SSS, SSP, ABAS, cortisol levels	NEPSY (Faces, ToM), cortisol (beginning of first and last, from pre-post for first and second rehearsal
Corbett et al. (126)	Social and others (stress levels)	W	W	Standardized scale, video coding, questionnaire	NEPSY, SRS, PSI, Parent/Child Dysfunction scale, ABAS, Companionship scale, PIP, cortisol levels	NEPSY (delayed memory for faces and memory for faces immediate v/s delayed), SRS (total, social awareness, social cognition), ABAS (home living, self-care), PSI (parent/child relationship), Cortisol (Theater 1 Camp—Play 2 and Theater Last Day—Play 2), Companionship scale (active involvement), NEPSY- (immediate memory for faces) (as)
Corbett et al. (165)	Social communication	B	B	Standardized scale, video coding, questionnaire	SRS, ABAS, NEPSY, PIP, ERP	NEPSY (MFD, MFI, TOM), ABAS (social- posttest), SRS (communication—at posttest and FUP), PIP (Group Play), ERP
Corbett et al. (87)	Social, behavioral, and others (stress levels)	B	B	Video coding, questionnaire	STAI-C, PIP, cortisol levels	STAI-C (trait), cortisol levels (beginning—end of first and middle days of intervention), PIP (as)
Ioannou et al. (125)	Social communication and others (anxiety levels)	B	B	Video coding, questionnaire	PIP, STAI- C	EG: PIP (solicited and unsolicited play) (as), STAI-C (trait anxiety)
Guli et al. (166)	Social communication, behavioral, and others (data collected on parent interview regarding efficacy of treatment)	B	B	Observational assessment, questionnaire, computerized tests	BASC, DANVA-2, observed social interactions	Social interaction—increase in positive interactions and decrease in solitary play
Kim et al. (167)	Social and behavioral	W	W	Observational assessment, questionnaire	RSE, EQ/SQ, resiliency scale, SCRETS	RSE (self-esteem−2 items), EQ/SQ (empathy−2 items), Resiliency scale (Comfort and Support from others−2 and 1 item, respectively, composite measures−3 items)
Reading et al. (168)	Social communication	B	B	Questionnaire	Rating of social behaviors	Scores on Social responsiveness, perspective of others, and participation and cooperation subscales increased from pretest to posttest in EG but not in CG.
Naniwadekar et al. (169)	Social communication	B	B	Questionnaire	ACPC-DD	ACPC-DD (social communication and emotion domains)
			W			EG and CG: ACPC-DD (social communication, and emotion domains)
Beadle-Brown et al. (170)	Social communication and behavioral	W	W	Standardized scale, questionnaire	ADOS-2, VABS-2, Ekman, parent/teacher rating of intervention	ADOS (reciprocal social interaction—module 3 (pre-post), total-module 3 (pre-post, pre-FUP, post-FUP), VABS (communication, socialization—pre-post), Ekman (pre-FUP) (pre-post- (as), ADOS-2 [total (pre-post and pre-FUP)] (as)
**Dance therapy**						
Arzoglou et al. (85)	Motor	B	W	Standardized scale	KTK (Korperkoordinationstest fur Kinder)	EG: KTK (total, backward walking, obstacle clearance on 1 leg, jumping sideways and sideways movement and repositioning), CG: No improvements
Koehne et al. (171)	Social and Behavioral	B	B	Standardized scale, video coding, questionnaire, computerized test	MET, IRI, Automation imitation paradigm, Finger tapping test of synchrony, ASIM	Emotion inference on MET, automatic imitation on automatic imitation paradigm, reduction in asynchrony on finger tapping test with virtual human-like partner, improvements in spontaneous imitation/synchronization and reciprocity/dialogue on ASIM in EG but not CG
Koch et al. (77)	Social, behavioral, and sensory	B	B	Video coding, questionnaire	HSI, QMT, SA-Q, EES-SF, FBT (Social skills)	All the variables except Empathy (EES-SF)
Hildebrandt et al. (172)	Behavioral	B	B	Standardized scale	SANS	SANS total score and all 5 subscales (affective blunting, alogia, avolition, anhedonia, attention)
Mastrominico et al. (173)	Behavioral	B	B	Questionnaire	CEEQ	–
Souza-Santos et al. (174)	Self-care skills and others (social participation and autism severity)	B	B	Standardized scale, questionnaire	CARS, FIM, WHODAS	CARS (all groups), WHODAS (dance+EAT)
			W			FIM—communication and psychosocial domains (dance), WHODAS (dance and dance+EAT)
Aithal et al. (175)	Social communication and behavior	B	B	Questionnaire	SCQ, SDQ	SCQ (social communication), SDQ (emotional social well-being)
**Miscellaneous: dance and music therapy**						
Mateos-Moreno & Atencia-Doña (176)	Social, behavioral, and motor	B	B	Standardized scale	ECA-R	ECA-R (overall score), factor 1 (interaction disorder), function of imitation, emotion, instinct and regulation/behavior variability disorders)

*EG, Experimental Group; CG, Control Group; ES, Effect Size; NA, Not Applicable; B, Between-group; W, Within-group; N, No; Y, Yes; CI, Confidence Interval; FUP, Follow-up; CRASS, Checklist for Communicative Responses/Acts Score Sheet; CGI-S, Clinical Global Impressions-Severity; CGI-I, Clinical Global Impressions-Improvement; BPRS, Brief Psychiatric Rating Scale; PDDBI-C; Pervasive Developmental Disorder Behavior Inventory-C; ESCS, Early Social Communication Scales; RJA, Responding to Joint Attention; IJA, Initiation of Joint Attention; CARS-BR, Childhood Autism Rating System-Brazilian version; IPR, Index of Peer Relations; RSES, Rosenberg Self-Esteem Scale; STAI-C, State Trait Anxiety Inventory (State and trait scales); VSEEC, Vineland Social-Emotional Early Childhood Scales; SRS, Social Responsiveness Scale; Mac-CDI, MacArthur Bates Communicative Development Inventories; PCRI, Parent-Child Relationship Inventory; MTDA, Music Therapy Diagnostic Assessment; ATEC, Autism Treatment and Evaluation Checklist; JA/ JTAT, Joint Attention; SSRS, Social Skills Rating System; RBS, Repetitive Behavior Scale; BOT-2, Bruininks-Oseretsky Test of Motor Performance-2nd Edition; FMCC, Fine Manual Control Composite; FMP, Fine Motor Precision; FMI, Fine Motor Integration; BCC, Body Coordination Composite; IPS, Interpersonal Synchrony; ADOS, Autism Diagnostic Observational Schedule; QOL, Quality Of Life; K-SSRS, Social Skills Rating System-Korean Version; SPRS, Social and Parent Relationship Scale; PANAS-C, Positive and Negative Affect Schedule for Children; MABC-2, Movement Assessment Battery 2nd Edition; SDQ, Strengths and Difficulties Questionnaire; AQR, Assessment of the Quality of the Relationship, DUACS, Duke University Autism Communication and Socialization; DCDQ, Developmental Coordination Disorder Questionnaire; AI, Attunement Index, TAI, Therapist Attunement Index. ARI-E2, Autism Research Institutes form E2 Checklist; ITB, Imitation Test Battery; RSB, Repetitive and Stereotyped Behavior Test Battery; GM, Gross Motor BASC-2, Behavior Assessment System for Children-Second Edition; ABC-C, Aberrant Behavior Checklist-Community; AQ, Autism Questionnaire; MAAS, Mindful Assessment and Awareness Scale; PSWQ, Penn State Worry Questionnaire; RRS, Ruminative Response Scale; WHO-5, World Health Organization-Five Well Being Index; FFMQ, Five Facet Mindfulness Questionnaire; IM-P, Interpersonal Mindfulness in Parenting Scale; PSI-C, Parenting Stress Index-Competence Scale; TSSA, Treatment and Research Institute for ASD Social Skills Assessment; MFMS, Modified Facial Mood Scale; EEG, Electroencephalogram; GARS, Gilliam Autism Rating Scale; NEPSY, Neuropsychological Measures; TOL, Tower of London; CCTT-T2, Children Color Trail Test; FPT, Five Point Test; BL, Bilateral; DSM, Diagnostic and Statistical Manual of Mental Disorders; BRIEF-2, Behavior Rating Inventory of Executive Function-2nd Edition; MVPA, Moderate to Vigorous Physical Activity; SSIS-RS, Social Skills Improvement System Rating Scale; GAS, Goal Attainment Scale; GEC, Global Executive Composite; EDI, Emory Dyssemia Index; BDI-Y, Beck Depression Inventory-Youth; CBCL, Child Behavior Checklist; DANVA-2, Diagnostic Analysis of Non-verbal Accuracy-2; SIOS, Social Interaction Observation System; MF, Memory for Faces; MFI, Memory for Faces Immediate; MFD, Memory for Faces Delayed; TOM, Theory of Mind; SSS, Stress Survey Schedule; SSP, Short Sensory Profile; ABAS, Adaptive Behavior Assessment System; PSI, Parent Stress Inventory; PIP, Peer Interaction Paradigm; ERP, Event Related Potential; EQ/SQ, Empathy/Systemizing Quotient; SCERTS, Social Communication, Emotional Regulation and Transactional Support Model; ACPC-DD, Activity Checklist for Preschool Children with Developmental Disability; VABS-2,Vineland Adaptive Behavior Scales; KTK, Korperkoordinationstest fur Kinder; MET, Multifaceted Empathy Test; IRI, Interpersonal Reactivity Index; ASIM, Assessment of Spontaneous Interaction in Movement; HIS, Heidelberg State Inventory; QMT, Questionnaire for Movement Therapy; SA-Q, Self-Awareness Questionnaire; EES, Emotional Empathy Scale; FBT, Fragebogen fuer Bewegungstherapie; SANS, Severity of Negative Symptoms; CEEQ, Cognitive and Emotional Empathy Questionnaire; CARS; Childhood Autism Rating System; FIM, Functional Independence Measure; WHODAS, WHO Disability Assessment Scale; ECA-R, Evaluation of Autistic Behavior-Revised Version; EAT, Equine Assisted Therapy; SCQ, Social Communication Questionnaire; SDQ, Strengths and Difficulties Questionnaire; STAI-C, State Trait Anxiety Scale for Children*.

**TABLE 4:** Study-wise list of reported and calculated effect sizes.

**References**	**Reported** **ES**	**Magnitude of** **reported ES**	**Type of** **effect calculated**	**Magnitude of** **calculated ES**	**CI range** **for ES**	**# of ES per measure where** **CI doesn't include 0**	**Agreement between reported and** **calculated ES**	**Comments**
**(A) Music/rhythm therapies**								
**Music/rhythm therapy**								
Edgerton et al. (129)	N	None	W	CRASS (3): 1.13–2.01	CRASS (3): 0.23–3.28	CRASS: 3 (total gain, musical vocal behavior, non-musical speech production)	NC	The paper reported significant findings for only total CRASS scores that was confirmed by our ES calculations. Non-parametric statistics used for all outcomes but no parameter estimates provided. Hence, ES calculated using provided Means and SDs
Hartshorn et al. (130)	N	None	B	Wandering (1): −1.28, Responding to touch negatively (1): 0.59, On-task passive behavior (1): 4.11, Resisting teacher (1): −1.57	Wandering (1): −1.77 to −0.78, Responding to touch negatively (1): 0.13–1.05, On-task passive behavior (1): 3.32–4.91, Resisting teacher (1): −2.08 to −1.06	Wandering: 1, Responding to touch negatively: 1, On-task passive behavior: 1, Resisting teacher: 1	NC	–
Boso et al. (131)	N	None	W	BPRS (3): −2.53 to −2.28, Music Skills (15): 0.16–2.66	BPRS (2): −4.54–0.63, Music Skills (15): −0.81–4.75	BPRS: 1 (T1–T2 and T1–T3), Music Skills: 3 (T1–T2 and T1–T3: singing a short melody, singing a long melody, playing the C scale on a keyboard)	NC	Non-parametric statistics used for all outcomes but no parameter estimates provided. Hence, ES calculated using provided Means and SDs
Kim et al. (119)	Y	PDDBI-C (clinician) (1): 0.79, ESCS (1): 0.97	B	ESCS (1): 1.91, Eye Contact (1): 4.06, Turn Taking (1): 4.06	ESCS (1): 0.41–3.41, Eye Contact (1): 1.89–6.23, Turn Taking (1): 1.89–6.23	ESCS: 1, Eye Contact: 1, Turn taking: 1	Fair	Calculated ES using *p*-values only
Kim et al. (101)	N	None	B	Joy (2): 0.47–0.55, Emotional Synchronicity (2): 0.51–0.54, Initiation of Engagement (1): 0.79, Initiation of Interaction (1): −0.30, Compliant Response (1): 0.11, No response (1): −0.64	Joy (2): −0.78–1.81, Emotional Synchronicity (2): −0.74–1.80, Initiation of Engagement (1): −0.49–2.08, Initiation of Interaction (1): −1.55–0.93, Compliant Response (1): −1.12–1.35, No response (1): −1.90–0.63	Joy: 0, Emotional Synchronicity: 0, Initiation of engagement: 0, Initiation of Interaction: 0, Compliant response: 0, No response: 0		
Gattino et al. (132)	Y	CARS-BR (1): −2.22	B	CARS-BR (1): −2.30	CARS-BR (3): −3.33 to −1.27	CARS-BR: 1 (non-verbal communication in autistic disorder)	Good	–
Hillier et al. (133)	N	None	W	IPR-participant (1): 0.42, IPR-parent (1): 0.33, RSES (1): 0.40, STAI-C (2): 0.45–0.48	NA	NA	NC	Non-parametric statistics used. ES calculated using *Z* values provided in the paper
Wan et al. (134)	N	None	W	IPA (1): 1.38–2.36	IPA (3): −0.33–4.92	IPA: 0	NC	Non-parametric statistics used for all outcomes but no parameter estimates provided. Hence, *p*-values used for ES calculations
Thompson et al. (135)	Y	VSEEC (1): 1.96	B	VSEEC (1): 1.97	VSEEC (1): 0.93–3.02	VSEEC: 1	Fair	–
	N	None	W	MTDA (1): −1.37	MTDA (1): −2.37 to −0.38	MTDA: 1		
LaGasse (136)	N	None	B	Eye gaze (1): 0.91, JA w/child (1): 1.24, JA w/adult (1): −0.02	Eye gaze (1): −0.08–1.91, JA w/child (1): 0.20–2.28, JA w/adult (1): −0.97–0.93	Eye gaze: 0, JA w/child: 1, JA w/adult: 0	NC	–
	N	None	W	SRS (1): −1.00	SRS (1): −2.00 to −0.01	SRS: 1	NC	
Ghasemtabar et al. (137)	N	None	B	SSRS-P (2): 0.47–0.59	SSRS-P (1): −0.29–1.36	SSRS-P: 0	NC	–
Srinivasan et al. (27)	Y	RMB (1): 0.5; Negative Affect (1): −0.32; Interested Affect (1); 0.43	W	RMB (2): −0.50 to −0.66; Negative Affect (1): −0.32; Interested Affect (1): 0.43	RMB (2): −1.37–0.18; Negative Affect (1): −0.97–0.32; Interested affect (1): −0.23–1.09	RMB: 0; Negative affect: 0; Interested affect:	Good	–
Srinivasan et al. (118)	Y	BOT (1): 0.6; Imitation (1): −0.65; IPS (1): 0.23	W	Imitation (1): −0.65; IPS (1): 0.23	Imitation (1): −1.35–0.05; IPS (1): −0.41–0.87	Imitation: 0; IPS: 0		
Srinivasan et al. (4)	Y	Social attention (3): 1.09–4.5; Spontaneous and responsive attention (6): 0.23–3.09	B	None	NA	NA		
	Y	JTAT (1): 0.55; Social Attention (9): 1.03–2.04	W	JTAT (1): 0.94	JTAT (1): 0.15–1.72	JTAT: 1		
Srinivasan et al. (6)	Y	Spontaneous verbalization (3): 0.51–0.61;	B	Spontaneous verbalization (1): 0.91	Spontaneous verbalization (1): 0.07–1.76	Spontaneous verbalization: 1	Calculated ES for JTAT and other outcome measures based on *p*-values reported in the paper. Hence, poor agreement between calculated and reported ES	
	Y	JTAT (1): 0.55; Response to social bids (2): 1.18–1.67; Verbalization with social partners (4): 0.67–1.07	W	JTAT (1): 0.94; Response to social bids (2): 1.20–1.70;	JTAT (1): 0.15–1.72 Response to social bids (2): 0.32–2.75;	JTAT: 1; Response to social bids: 2		
Bieleninik et al. (116)	N	None	B	ADOS (6): −0.03–0.2; SRS (6): −0.04–0.02	ADOS (6): −0.25–0.44; SRS (6): −0.28–0.26	ADOS: 0, SRS: 0	NC	No significant differences found in ADOS and SRS scores from baseline to post-test or at FUP
Mossler et al. (117)	N	None	W	Couldn't calculate ES	NA	NA	NA	–
Dvir et al. (98)	N	None	W	AI (1): −0.26, TAI (1): −0.27, ADOS-2 (1): 0.20	ADOS-2 (1): −0.13–0.54	ADOS-2: 0	NC	Non-parametric statistics i.e., Wilcoxon's signed rank test used and *Z*-scores reported. Thus, the Kerby's formula of r = Z/(sqrt)N was used (*N* = number of observations made and r = ES estimate)
Yoo & Kim (138)	N	None	W	SRS-K (3): 1.35–1.63	SRS-K (3): 0.12–3.07	SRS-K: 3 (total, cooperation and self-control)	NC	Non-parametric statistics used for all outcomes but no parameter estimates provided. Hence, ES calculated using provided Means and SDs
Willemin et al. (139)	Y	Smiley 8 (1): −0.25, Fun-O-Meter (1): −0.36	W	Smiley 8 (2): −0.55, Fun-O-Meter (2): −0.56	Smiley 8 (2): −1.17–0.06, Fun-O-Meter (2): −1.18–0.06	Smiley 8: 0, Fun-O-meter: 0	Good agreement between ES calculated through the *p* and *F* values provided. But poor agreement between calculated and reported ES	ES calculated and triangulated using p and *t*-values
Lowry et al. (140)	N	None	B	SDQ (3): 0.73–0.90	NA	NA	NC	Non-parametric statistics (Mann-Whitney *U*-test) used and *U*-values were provided in the paper. ES calculation based on formula, ES = U/n1+n2 (n1 and n2 = sample sizes of the 2 Groups being compared)
Stephen (141)	N	None	B	SSRS (1): 0.60	SSRS (1): −0.12–1.34	SSRS: 0	NC	–
Schmid et al. (142)	N	None	W	DUACS (1): 0.78, PDDBI subscales (7): −0.23–0.23	DUACS (1): 0.49–1.07, PDDBI subscales (7): −1.51–0.48	DUACS: 1, PDDBI subscales: 1 (associative learning skills)	NC	–
Rabeyron et al. (143)	Y	CGI (1): −0.8, CARS (1): 0.22, ABC (3): −0.31–0.02	B	CGI (1): −1.05, CARS (1): −0.13, ABC (3): −0.46 to −0.26	CGI (1): −1.74 to −0.35, CARS (1): −0.79–0.52, ABC (3): −1.12–0.39	CGI: 1, CARS: 0, ABC: 0	Poor	–
Cibrian et al. (144)	N	None	B	Couldn't calculate ES	NA	NA	NA	–
**(B) Yoga/mindfulness and martial arts-based interventions**								
**Yoga/mindfulness-based therapy**								
Radhakrishna et al. (120)	N	None	W	None	NA	NA	NA	NA
Radhakrishna et al. (121)	N	None	W	None	NA	NA	NA	NA
Rosenblatt et al. (145)	N	None	W	BASC (2): 0.43–0.49; Aggregate BASC and ABC (4): 0.60–0.88	BASC (2): 0.050–19.83; Aggregate BASC and ABC (4): 0.01–1.52	BSI: 2 (BSI, atypicality); Aggregate BASC and ABC: 4 (irritability-atypicality, irritability-BSI, irritability-depression, irritability- externalization	NC	Study conducted overall sample analysis as well as sub-group analysis for 5–12-year-olds. BASC improvements were seen in analysis for total sample and the sub-group. Aggregate of ABSC and ABC improved in sub-group only.
Koenig et al. (88)	Y	Teacher ABC (4): 0.53–1.19	B	Teacher ABC (4): 0.50–0.65	Teacher ABC (4): −0.08–1.24	Teacher ABC: 1 [total (*F*-value)]	Poor agreement for teacher-ABC (lethargy, total), good agreement for teacher- ABC (irritation, stereotypic behavior, hyperactivity, inappropriate speech)	ES calculated and triangulated using *p* and *F* values
de Bruin et al. (146)	Y	WHO (2): 0.55–0.63; RRS (1): −0.92; SRS (4): −0.4−0.17;	W	Couldn't calculate ES	NA	NA	NA	-
Narasingharao et al. (147)	N	None	W	EG: SQ (15): 0.28–0.64, FQ (16): 0.48–0.63, BQ (29): 0.50–0.62	NA	NA	NA	Non-parametric statistics i.e., Wilcoxon's signed rank test used and *Z*-scores reported. Thus, the Kerby's formula of r = Z/(sqrt)N was used (*N* = number of observations made and r = ES estimate)
Sotoodeh et al. (148)	N	None	B	ATEC (4): 1.42–2.66	ATEC (4): 0.39–3.93	ATEC: 4 (sociability, sensory/cognitive/ awareness, health/physical/behavior and total)	NC	ES calculated and triangulated using *p* and *F* values
Litchke et al. (149)	N	None	W	TSSA (4): −3.04–0.92	TSSA (4): −7.17–2.64	TSSA: 0	NC	ES calculated and triangulated using *t-* and *p*-values except for Total scores where Means and SD were provided
Kaur & Bhat (150)	N	None	B	None			Poor	
	Y	EG: BOT-2 (1): 0.56, % Imitation (2): 0.96–1.48, CG: BOT-2 (2): 0.3–0.32, % Imitation (1): 0.83	W	EG: BOT-2 (1): 0.96, % Imitation (2): 0.76–0.76, CG: BOT-2 (2): 0.66–0.66, % Imitation (1): 0.77	EG: BOT-2 (1): 0.12–1.81, % Imitation (2):−0.011 to 1.55, CG: BOT-2 (2):−0.04–1.37, % Imitation (1):−0.011–1.55	EG: BOT-2: 1 (Bilateral coordination), % Imitation: 0, CG: BOT-2: 0, % Imitation: 0		*p*-values used for calculation of ES
Vidyashree et al. (151)	N	None	B	Couldn't calculate ES	NA	NA	NA	NA
	N	None	W	Couldn't calculate ES	NA	NA		
Tanksale et al. (152)	Y	BRIEF-2 (10): −0.69 to −0.37, Children's sleep habits questionnaire (4): −0.48–0.33, Emotion awareness (3): −0.52–0.59, Anxiety (1): −0.43	B	BRIEF-2 (10): −0.64 to −0.34, Children's sleep habits questionnaire (4): 0.5–0.59, Emotion awareness (3): 0.51–0.73, Anxiety (1): 0.56	BRIEF-2 (10): −1.16–0.15, Children's sleep habits questionnaire (4): −0.004–1.1, Emotion awareness (3): 0.002–1.25, Anxiety (1): 0.05–1.08	BRIEF-2: 2 (FUP: GEC, self-monitor) Children's sleep habits questionnaire: 3 (bedtime resistance, sleep onset delay, sleep breathing disorder), Emotion awareness: 3, Anxiety: 1	Good	–
**Martial Arts**								
Bahrami et al. (122)	N	None	B	GARS (2): −0.66 to −0.47	GARS (2): −0.14–0.24	GARS: 0	NC	Paper reported significant within-group effects confirmed by our ES calculations. Between-group comparisons were not significant
	N	None	W	GARS (2): −0.73 to −0.60	GARS (2): −1.35 to −0.003	GARS: 2 (Stereotypy: pre-post and pre-FUP)		
Movahedi et al. (153)	N	None	B	GARS (2): −1.10 to −0.78	GARS (2): −1.93–0.01	GARS: 1 (Social Interaction: pre-post)	NC	
	N	None	W	GARS (2): −1.13 to −0.82	GARS (2): −1.93 to −0.11	GARS: 2 (Social Interaction: pre-post and pre-FUP)		
Bahrami et al. (86)	N	None	B	GARS (2): −0.75 to −0.64	GARS (2): −1.49–0.090	GARS: 1 (Communication: pre-post)	NC	
	N	None	W	GARS (2): −0.81 to −0.70	GARS (2): −1.45 to −0.07	GARS: 2 (Communication: pre-post and pre-FUP)		
Chan et al. (154)	Y	Neuropsychological measures (1): 0.84, Custom questionnaire (3): 0.86	B	Neuropsychological measures (1): 0.20, Custom questionnaire (1): 0.73	Neuropsychological measures (1):−0.42–0.822, Custom questionnaire (2): 0.09–1.37	Neuropsychological measures: 0, Custom questionnaire: 1 (temper outburst (*t*-value)	Between-group ES show good agreement. Within-group ES for EG show poor agreement	Paper reported only within-group effects. ES calculated and triangulated using *p* and *t* values
	Y	Neuropsychological measures (2): 0.80–0.83, ATEC (3): 0.2 −0.68	W	Neuropsychological measures (2):−0.4–0.57, ATEC (3):−0.33 to −0.25, ACC (2): 0.06	Neuropsychological measures (2):−0.88–1.07, ATEC (3):−0.82–0.22, ACC (2):−0.39–0.52	Neuropsychological measures: 1 (FPT), ATEC: 0, ACC: 0		
Chan et al. (155)	Y	Memory (2): 0.57–0.73	W	Memory (2): 0.55 −0.70; EEG (6): 0.42–0.68	Memory (2): 0.02–1.26: EEG (6):−0.03–1.24	Memory: 2; EEG: 4	Good agreement for memory scores	ES calculated and triangulated using *p* and *t* values
Figueiredo et al. (156)	N	None	W	SDQ (1): 0.87, Connors scale for parents (3): 0.87–0.97	SDQ (1):−0.16–1.90, Connors Scale for Parents (3):−0.15–2.04	SDQ: 0, Connor's Scale: 0	NC	Non-parametric statistics used for all outcomes but no parameter estimates provided. Hence, *p*-values used for ES calculations
Kim et al. (157)	Y	Single Leg R (eyes closed) (1): 0.5	B	Single Leg R (eyes closed) (1): Could not calculate	NA	NA	NC	The SD for control group was reported as 0 in the original paper; hence ES could not be computed
	N	None	W	Single leg L (eyes open) (1): −0.522	Single leg L (eyes open) (1): −1.42–0.37	Single leg L (eyes open): 0		
Phung & Goldberg (104)	Y	Hearts and flowers accuracy % (2): 0.83–1.01, BRIEF-2 (2):−0.88 to −0.67	B	Hearts and flowers accuracy % (2): 0.442–0.72, BRIEF-2 (3):−0.55 to −0.45	Hearts and flowers accuracy % (2):−0.24–1.42, BRIEF-2 (2):−2.25–0.23	Hearts and flowers accuracy %: 1 (congruent), BRIEF-2: 0	Poor	–
Phung et al. (123)	Y	SSIS (2): −1.61–1.19	B	SSIS (2): −1.13–0.63	SSIS (2): −1.86–1.33	SSIS: 1 (problem behaviors)	Fair	
Sarabzadeh et al. (158)	N	None	B	MABC-2 (3): −3.61 to −3.14	MABC-2 (3): −5.11 to −1.76	MABC: 3 (total, ball skills and balance)	NC	Paper reported significant between- and within-group effects. We report only between-group ES
Garcia et al. (127)	Y	MVPA (% activity) (1): 0.97	W	MVPA (% activity) (1): 0.54	MVPA (% activity) (1): −0.076–1.16	MVPA (% activity): 0	Poor	Non-parametric statistics used for all outcomes but no parameter estimates provided. Hence, *p*-values used for ES calculations
Rivera et al. (128)	N	None	W	ABD (6): −0.29–0.01	ABC (6): −0.71–0.42	ABC: 0	NC	No improvements reported by paper, confirmed by our ES calculations
Ansari et al. (159)	N	None	B	Static balance (1): 2.3, dynamic balance (1): 5.34	Static balance (1): 1.17–3.44, dynamic balance (1): 3.46–7.21	Static balance: 1, dynamic balance: 1	NC	–
AdibSaber et al. (160)	N	None	B	Sleep questionnaire (6): −3.87 to −1.41	Sleep questionnaire (6): −5.35 to −0.43	Sleep questionnaire: 6 (total score, sleep duration, sleep anxiety, night time awakening, parasomnia, daytime sleepiness)	NC	–
Greco & de Ronzi (161)	Y	SSIS-RS (2): 2.64–2.85, BRIEF (4): 0.97–1.63	B	SSIS-RS (2):−0.92–1.15, BRIEF (4):−0.46 to −0.33	SSIS-RS (2):−1.7–1.95, BRIEF (4):−4.19–0.41	SSIS-RS: 1 (social skills)	Poor.	Reported ES larger in magnitude than calculated ES.
Tabeshian et al. (162)	N	None	B	GARS (1): −0.49	GARS (1): −1.17–0.17	GARS: 0	NC	–
**(C) Theater/dramatic, dance-based, and miscellaneous interventions**								
**Theater/dramatic training**								
Lerner et al. (163)	Y	None	B	Couldn't calculate ES	NA	NA	NA	–
	N	None	W	Couldn't calculate ES	NA	NA		
Lerner & Mikami (164)	Y	SIOS (2):−0.98 to −1.17, SSRS-T (1): 0.59	B	SIOS (2):−2.02 to −1.88, SSRS-T (1): 0.33	SIOS (2):−2.36−0.57, SSRS-T (1):−0.76–1.43	SIOS: 0, SSRS-T: 0	Poor	–
	Y	CG: Sociometrics (1): 0.7	W	CG: Sociometrics (1): 1.16	CG: Sociometrics (1): −0.38–2.72	Sociometrics: 0		
Corbett et al. (124)	Y	NEPSY (2): 1.44–1.68	W	NEPSY (2): 0.35–0.429, Cortisol (3): −2.55 to −1.69	NEPSY (2):−0.50–1.30, Cortisol (3):−4.55 to −0.22	NEPSY: 0, Cortisol (3): 3	Poor	Significant differences found between cortisol levels measured before and after the first and middle rehearsals and also those taken at the beginning of D1 and D3 sessions
Corbett et al. (126)	Y	NEPSY (2): −0.99 to −0.89, SRS (3): 0.23–1.46, ABAS (2): −0.34 to −0.29, PSI (1): 0.71, Cortisol (5): −0.72–1.24	W	NEPSY (2): 0.78–0.89, SRS (3):−3.30 to −0.26, ABAS (2): 0.31–0.36, PSI (1):−0.60, Companionship scale (1): 0.56, Cortisol (5): 0.73–0.84	NEPSY (2): 0.002–1.72, SRS (3):−3.27–0.4, ABAS (2):−0.36–1.05, PSI (1):−1.34–0.13, Companionship scale (1):−0.16–1.29, Cortisol (5):−0.03–1.65	NEPSY: 2 (MFD and MFD), SRS: 1 (social cognition), ABAS: 0, PSI: 0, Companionship scale: 0, Cortisol: 3	Good agreement overall except for ABAS communication and play-based cortisol levels	Non-parametric statistics used for all outcomes but no parameter estimates provided. Hence, *p*-values used for ES calculations for cortisol levels measured at different times. Reductions in cortisol levels during play between D1 and D2 and between D2 to end of training
Corbett et al. (165)	N	None	B	ABAS (1): 0.75, SRS (2):−0.83, PIP (1): 0.74, NEPSY (3): 0.73 −0.97, ERP (1): 0.90	ABAS (1): 0.004–1.49, SRS (2):−2.58 to −0.08, PIP (1): 0.002–1.49, NEPSY (3):−0.01–1.73, ERP (1): 0.15–1.66	ABAS: 1 (social), SRS: 1 (communication), PIP: 1 (group play), NEPSY: 2 (MFD, TOM), ERP: 1 (social brain)	Good agreement for all except SRS communication	–
Corbett et al. (87)	N	None	W	STAI-C (1): −0.49, Cortisol (2): −0.61 to −0.58	STAI-C (1): −1.04–0.04, Cortisol (2): −1.17 to −0.02	STAI-C: 0, Cortisol: 2	NC	Reductions in cortisol levels during play from beginning to the end of first and middle days of intervention
Ioannou et al. (125)	N	None	B	PIP (4): 0.47–0.66, STAI-C (2): 0.02–0.63	PIP (4): 0.01–1.14, STAI-C (2): −0.44–1.11	PIP: 4 (solicited and unsolicited group and self-play), STAI-C: 1 (trait anxiety)	NC	–
Guli et al. (166)	Y	None	B	Observed behaviors (2): −0.54–0.68	Observed behaviors (2): −1.59–1.66	Observed behaviors: 0	NC	Non-parametric statistics used for all outcomes but no parameter estimates provided. Hence, Means and SDs used for ES calculations
Kim et al. (167)	N	None	W	RSE (2): −0.47–0.5, EQ/SQ (2): 0.30–0.40, Comfort with others (2): 0.75 −0.4, Support from others (1): −0.46, Composite measures (3): 0.34–0.58	RSE (2): −1.01–1.02, EQ/SQ (2): −0.20–0.91, Comfort with others (2): −0.11–1.31, Support from others (1): −0.98–0.05, Composite measures (3): −0.16–1.12	RSE: 0, EQ/SQ: 0, Comfort with others: 1 (I can meet new friends easily), Support from others: 0, Composite measures: 2 (self-esteem and comfort with others)	NC	Non-parametric statistics used for all outcomes but no parameter estimates provided. Hence, Means and SDs used for ES calculations
Reading et al. (168)	N	None	B	Couldn't calculate ES	NA	NA	NC	Main effect of group X time provided, thus ES could not be calculated
Naniwadekar et al. (169)	N	None	B	Communication (1): −1.01	Communication (1): −2.49–0.45	Communication: 0	NC	–
	N	None	W	Communication (1): 1.07	Communication (1): −3.50–0.19	Communication: 1		
Beadle-Brown et al. (170)	Y	ADOS-2 (1): 1.96, VABS (2): 3.42–6.07, Ekman (1): 2.12	W	ADOS-2 (4): 0.17–0.55, VABS (2): 0.51–0.62	NA	NA	Poor	Non-parametric statistics i.e., Wilcoxon's signed rank test used and *Z*-scores reported. Thus, the Kerby's formula of r = Z/(sqrt)N was used (*N* = number of observations made and r= ES estimate)
**Dance therapy**								
Arzoglou et al. (85)	N	None	B	KTK (5): 0.88–2.63	KTK (5): −0.32–4.32	KTK: 0	NC	Paper reported significant differences between groups, but ES calculations suggest no significant between or within-group differences
Koehne et al. (171)	Y	None	B	MET (1): 0.31, AIP (1): 0.56, asynchrony test (1): −0.42, ASIM (2): 0.71–1.21	MET (1): −0.23–0.866, AIP (1): −0.004–1.12, asynchrony test (1): −1.106–0.26, ASIM (2): −0.12–2.1	MET: 0, AIP: 0, asynchrony test: 0, ASIM: 1 (reciprocity/dialogue	Fair	–
	N	MET (1): 0.58, AIP (1): 0.47, Asynchrony test (1): −0.63, ASIM (2): 1.27–1.25	W	MET (1): 0.76, AIP (1): 0.42, Asynchrony test (1): −0.34, ASIM (2): 0.41–1.28	MET (1): −0.11–0.68, AIP (1): −0.341–0.44, Asynchrony test (1): −0.83–0.14, ASIM (2): −0.13–0.96	MET: 0, AIP: 0, asynchrony test: 0, ASIM: 1 (reciprocity/dialogue)		
Koch et al. (77)	Y	QMT (1): 0.62, SOA (1): 0.72, HSI (1): 0.68, FBT (1): 0.67	B	QMT (1): 0.59, SOA (1): 0.63, HSI (1): 0.68, FBT (1): 0.54	QMT (1): −0.12–1.31, SOA (1): −0.09–1.35, HSI (1): −0.04–1.40, FBT (1): −0.16–1.26	QMT: 0, SOA: 0, HSI: 0, FBT: 0	Fair	–
Hildebrandt et al. (172)	N	None	B	SANS (6): −0.47 to −0.01	SANS (6): −0.97–0.48	SANS: 0	NC	The study had a lot of missing data but ES calculations using full sample vs. completed cases only did not reveal substantial differences in magnitude and direction of ES and their CIs. Hence, estimates from full sample are reported
Mastrominico et al. (173)	N	None	B	IRI/SPF-E (1): 0.03, CEEQ (6): −0.17–0.23	IRI/SPF-E (1): −0.58–0.57, CEEQ (6): −0.71–0.78	IRI/SPF-E (1): 0, CEEQ (6): 0	NC	No improvements reported by paper, confirmed by our ES calculations
Souza-Santos et al. (174)	N	None	B	CARS (1): −2.63 (dance-EAT), –d.36 (dance-dance and EAT), 1.26 (EAT-dance and EAT)	CARS (1): −3.60 to −1.65 and –n.15 to −0.56 and 0.48–2.05	CARS: 1 (dance, equine and dance+equine)	NC	Non-parametric statistics used for all outcomes but no parameter estimates provided. Hence, *p*-values used for ES calculations for WHODAS and FIM
	N	None	W	CARS (1): −4.72 (dance-EAT), –d.72 (dance-dance and EAT), −2.09 (EAT-dance and EAT), FIM (2): 0.64–0.73, WHODAS (1): 0.91	CARS (1): −6.82 to −1.04, FIM (2): 0.02–1.35, WHODAS (1): 0.24–1.59	CARS: 1 (between all 3 groups), FIM: 2 (communication, psychosocial adjustments in dance group), WHODAS: 1 (dance+equine)		
Aithal et al. (175)	Y	SCQ (2): 0.09–1.523, SDQ (2): 0.02–1.127	B	SCQ (2): 0.33–0.48, SDQ (2): 0.22–0.68	SCQ (2): −1.28–0.45, SDQ (2): −1.49–0.56	SCQ: 0, SDQ: 0	Poor	
**Miscellaneous: dance and music therapy**								
Mateos-Moreno & Atencia-Doña (176)	Y	Overall score on ECA-R (1): 2.04, Interaction disorder (1): 1.18, Function of imitation (1): 2.35, Function of emotion (1): 1.41, Function of instinct (1): 1.88, Function of behavior (1): 2.37	B	Interaction disorder (1): 0.5, Function of imitation (1): 0, Function of emotion (1): 1, Function of instinct (1): 0.5, Function of behavior (1): 0	NA	NA	Poor	Non-parametric statistics i.e., Wilcoxon's signed rank test used and *Z*-scores reported. Thus, the Kerby's formula of r = Z/(sqrt)N was used (*N* = number of observations made and r = ES estimate)

*Effect size (ESs) have been calculated and reported only for variables and measures where significant effects were reported in the original study. Effect sizes have been reported as absolute values in terms of magnitude only (for instance, for some variables a negative ES implies improvement. The numbers in parentheses reported next to the measure in columns 4–6 indicate the number of effect sizes calculated per measure. In case of multiple effect sizes calculated per measure, an ES range has been reported. Whenever possible, an attempt was made to triangulate ES values (for instance, if the study provide p-, t-, and/or F-values, ESs were calculated using all methods and checked for agreement). In addition, we have also reported Confidence intervals (CI) for effect sizes. In cases where multiple effect sizes have been calculated per outcome measure, CI ranges have been reported. Lastly, we have also mentioned the number of statistically significant effect sizes (CI do not include 0) per outcome measure for each study. In papers that used multiple control groups, results are reported only from the ASD groups that received creative movement interventions*.

*EG, Experimental Group; CG, Control Group; ES, Effect Size; NA, Not Applicable; NC, No Comparison Possible Since Original Paper did not report effect size estimates; B, Between-group; W, Within-group; N, No; Y, Yes; CI, Confidence Interval; FUP, Follow-up; CRASS, Checklist for Communicative Responses/Acts Score Sheet; CGI-S, Clinical Global Impressions-Severity; CGI-I, Clinical Global Impressions-Improvement; BPRS, Brief Psychiatric Rating Scale; PDDBI-C, Pervasive Developmental Disorder Behavior Inventory-C; ESCS, Early Social Communication Scales; RJA, Responding to Joint Attention; IJA, Initiation of Joint Attention; CARS-BR, Childhood Autism Rating System-Brazilian version; IPR, Index of Peer Relations; RSES, Rosenberg Self-Esteem Scale; STAI-C, State Trait Anxiety Inventory (State and Trait scales); VSEEC, Vineland Social-Emotional Early Childhood Scales; SRS, Social Responsiveness Scale; Mac-CDI, MacArthur Bates Communicative Development Inventories; PCRI, Parent-child Relationship Inventory; MTDA, Music Therapy Diagnostic Assessment; ATEC, Autism Treatment and Evaluation Checklist; JA/ JTAT, Joint Attention; SSRS, Social Skills Rating System; RBS, Repetitive Behavior Scale; BOT-2, Bruininks-Oseretsky Test of Motor Performance-2nd Edition; FMCC, Fine Manual Control Composite; FMP, Fine Motor Precision; FMI, Fine Motor Integration; BCC, Body Coordination Composite; IPS, Interpersonal Synchrony; ADOS, Autism Diagnostic Observational Schedule; QOL, Quality Of Life; K-SSRS, Social Skills Rating System-Korean Version; SPRS, Social and Parent Relationship Scale; PANAS-C, Positive and Negative Affect Schedule for Children; MABC-2, Movement Assessment Battery 2nd Edition; SDQ, Strengths and Difficulties Questionnaire; AI, Attunement Index, TAI, Therapist Attunement Index; DUACS, Duke University Autism Communication and Socialization; ARI-E2, Autism Research Institutes form E2 Checklist; ITB, Imitation Test Battery; RSB, Repetitive and Stereotyped Behavior Test Battery; GM, Gross Motor BASC-2, Behavior Assessment System for Children-Second Edition; ABC-C, Aberrent Behavior Checklist-Community; AQ, Autism Questionnaire; MAAS, Mindful Assessment and Awareness Scale; PSWQ, Penn State Worry Questionnaire; RRS, Ruminative Response Scale; WHO-5, World Health Organization-Five Well Being Index; FFMQ, Five Facet Mindfulness Questionnaire; IM-P, Interpersonal Mindfulness in Parenting Scale; PSI-C, Parenting Stress Index-Competence Scale; TSSA, Treatment and Research Institute for ASD Social Skills Assessment; MFMS, Modified Facial Mood Scale; EEG, Electroencephalogram; GARS, Gilliam Autism Rating Scale; NEPSY, Neuropsychological Measures; TOL, Tower of London; CCTT-T2, Children Color Trail Test; FPT, Five Point Test; BL, Bilateral; DSM, Diagnostic and Statistical Manual of Mental Disorders; BRIEF-2, Behavior Rating Inventory of Executive Function-2nd Edition; MVPA, Moderate to Vigorous Physical Activity; SSIS-RS, Social Skills Improvement System Rating Scale; GEC, Global Executive Composite; EDI, Emory Dyssemia Index; BDI-Y, Beck Depression Inventory-Youth; CBCL, Child Behavior Checklist; DANVA-2, Diagnostic Analysis of Non-verbal Accuracy-2; HSI, Heidelberger State Inventory; SIOS, Social Interaction Observation System; SSRS-T, SSRS Teacher Version; MF, Memory for Faces; MFI, Memory for Faces Immediate; MFD, Memory for Faces Delayed; TOM, Theory of Mind; SSS, Stress Survey Schedule; SSP, Short Sensory Profile; ABAS, Adaptive Behavior Assessment System; PSI, Parent Stress Inventory; PIP, Peer Interaction Paradigm; ERP, Event Related Potential; EQ/SQ, Empathy/Systemizing Quotient; SCERTS, Social Communication, Emotional Regulation and Transactional Support Model; ACPC-DD, Activity Checklist for Preschool Children with Developmental Disability; VABS-2,Vineland Adaptive Behavior Scales; KTK, Korperkoordinationstest fur Kinder; MET, Multifaceted Empathy Test; IRI, Interpersonal Reactivity Index; ASIM, Assessment of Spontaneous Interaction in Movement; HIS, Heidelberg State Inventory; QMT, Questionnaire for Movement Therapy; SA-Q, Self-awareness Questionnaire; EES, Emotional Empathy Scale; FBT, Fragebogen fuer Bewegungstherapie; SANS, Severity of Negative Symptoms; CEEQ, Cognitive and Emotional Empathy Questionnaire; CARS; Childhood Autism Rating System; FIM, Functional Independence Measure; WHODAS, WHO Disability Assessment Scale; EAT, Equine Assisted Therapy; ECA-R, Evaluation of Autistic Behavior-Revised Version; SCQ, Social Communication Questionnaire*.

#### Social Communication

A total of 47 studies i.e., 17 Level I (7 music, 1 yoga, 3 martial arts, 4 theater, 2 dance), 15 Level II studies (6 music, 1 yoga, 2 martial arts, 4 theater, 1 dance, 1 music and dance combined) and 15 Level III studies (7 music, 2 yoga, 2 martial arts, 4 theater) assessed changes in social communication skills following CMT (refer to section Risk of Bias Assessment for definition of levels; see **Table 6**). The social communication outcome measures employed included standardized tests such as the ADOS, CARS, and GARS, observational measures such as the ESCS and JTAT, parent/teacher-report questionnaires such as the ATEC, SRS, VABS, and SSRS, as well as video-based coding measures of joint attention, verbalization, and turn taking (see Tables 3A–C). All except one Level II study (121) reported quantitative data on social communication outcomes following CMT interventions. Using data from original reports, we were able to calculate a total of 91 ES, specifically, 38 ES from 12 out of the total 17 level I studies, 21 ES from 11 out of total 15 level II studies, and 32 ES from 9 out of the total 15 level III studies.

Of the 17 Level I studies, three studies reported no significant changes (1 martial arts, 1 theater, 1 dance) and 14 studies (7 music, 1 yoga, 2 martial arts, 3 theater, 1 dance) reported positive effects with small to large effect sizes (ES: 0.09–4.06) within their original report. Out of the 38 ES we calculated, CI for 14 ES from 9 studies did not include 0 (5 Music, 1 yoga, 2 martial arts, 1 theater). The largest multi-site study in our review that included 364 children from 9 countries was the only study that reported only small improvements on the social affect sub-domain of the ADOS (ES: 0.03–0.2) and the social motivation and autistic mannerisms subscales of the SRS (ES: 0.04–0.02) in the experimental group following a 20-week improvisational music therapy program compared to a comparison group that received a standard-of-care intervention; however, these findings were not statistically significant at the between-group level (116).

Among all 15 Level II studies, improvements of medium sizes (ES: 0.22–0.79) in social communication outcomes were reported in the original papers by music (6), yoga (1), martial arts (2), theater (4), dance (1) and combined music and dance (1) interventions, but out of the 21 ES we calculated, only 6 ES from 3 studies (2 martial arts, 1 theater) were statistically significant (CI did not include 0). In terms of the level III studies, only 3 of the 15 studies reported small to large ES on the ADOS and SRS following theater (2) and yoga (1) interventions (see Tables 4B,C). Similarly, despite large mean ES (0.88–3.04) estimates, only 6 out of the 32 ES we calculated from the 3 Level III studies did not include 0 (2 music and 1 theater).

Overall, out of the 91 calculated ES across 47 studies, 26 ES from 15 studies (~32% studies) were statistically significant (CI did not include 0) and indicated effects that were varying in magnitude from small to large (see Tables 4A–C, **6**). Specifically, there is moderately strong evidence for beneficial effects of music (5 Level I and 2 Level III studies) followed by martial arts (2 Level I and Level II studies each), with limited insufficient evidence for yoga (1 Level I study), and theater (1 Level I, 1 Level II, and 1 Level III study) interventions in promoting social communication outcomes (see **Table 6**). One salient finding from our ES calculations is that although all 12 theater studies assessed social communication skills, only 3 of these studies (25%) showed significant improvement (CI of ES did not include 0) in social communication despite the heavy emphasis on peer-mediated social skill training in theater-based interventions (see **Table 6**). Taken altogether, our review suggests that there is *moderate evidence* from multiple Level I studies for *small-to-large-sized improvements* in *social communication* skills following mainly music and martial arts-based approaches.

#### Behavioral—Affective

Twenty-one papers in our review assessed *behavioral* outcomes i.e., 8 Level I (2 music, 1 yoga, 1 martial arts, 3 theater, 1 dance), 9 Level II studies (3 music, 2 yoga, 1 martial art, 1 theater, 1 dance, and 1 combined dance and music), and 4 Level III studies (1 yoga, 1 martial arts, and 2 theater). Behavioral states were assessed using standardized scales such as the GARS, Scale for Assessment of Negative Symptoms (SANS), and Pervasive Developmental Disorder Behavioral Inventory-Children (PDDBI-C), video-based coding of on- and off- task behaviors and the amount of redirection required during training sessions, as well as using questionnaire-based measures such as the Child Behavior Checklist (CBCL), Aberrant Behavior Checklist (ABC-C), and Autism Treatment and Evaluation Checklist (ATEC) (see Tables 3A–C). A total of 18 studies reported significant effects of CMT on behavioral skills, with the remaining 3 theater-based studies (87, 124, 166) reporting non-significant effects. Only 6 of the 18 studies that reported positive effects of CMT provided ES estimates in their original report ranging from small to large in magnitude. Furthermore, we were able to calculate a total of 39 ES from 14 studies, specifically, 8 ES from 5 out of the total 8 Level I studies, 20 ES from 6 out of the total 9 Level II studies, and 11 EFs from 3 out of the total 4 Level III studies. Our own calculations based on these papers suggested mostly medium effects for CMT (note that we obtained large ES estimates for 2 studies where *F-*values were used to calculate ES; however, these measures are more imprecise compared to ES calculations using means and SD/SE values).

As an example of positive intervention effects, Hartshorn et al. (130) reported significant, large improvements in on-task behaviors (ES: 1.28–4.11) from an early to a late training session following their Level II 8-week music and movement intervention compared to a no-intervention control group (130). Similarly, following a Level II, 14-week, Kata martial art intervention, the experimental group showed significant, medium-sized improvements on the stereotypy subscale (ES: 0.47–0.66) of the GARS compared to a control group, with gains retained at 1 month follow-up (122). Hildebrandt et al. conducted a Level II RCT to assess the effectiveness of a 10-week manualized dance movement therapy intervention on negative symptoms using the standardized, clinician-rated SANS scale in 78 individuals with ASD. The authors concluded that although the results did not reach statistical significance at the between-group level, they found promising trends for greater symptom reduction (ES: 0.008–0.47) in the experimental group compared to the waitlist control group in overall negative symptoms as well as most subscales of the SANS (177). Overall, out of the 39 total calculated ES across 14 studies, 17 estimates calculated from 7 studies (~50% studies), specifically, 2 music (Level II), 3 yoga (1 each of Levels I, II, and III), 1 martial art (Level III), and 1 theater-based (Level I) intervention were statistically significant (CI did not include 0). Although the present state of the literature is insufficient to systematically evaluate the differing effects of various types of CMT, there is currently *some consistent evidence* for *medium-sized, positive effects* of CMT in reducing *behavioral* symptoms in individuals with ASD.

A total of 20 studies, i.e., 5 Level I (1 music, 3 theater, 1 dance), 6 Level II (1 music, 1 yoga, 1 theater, 2 dance, 1 music and dance combined), and 9 Level III (3 music, 2 yoga, 4 theater) studies assessed *affective* outcomes using questionnaires such as the Empathy/Systemizing Quotient (EQ/SQ), Brief Psychiatric Rating Scale (BPRS) Positive and Negative Affect Schedule (PANAS-C), and a computerized test such as the Multifaceted Empathy Test (MET) (see Tables 3A–C). We found that only 4 out of the 20 studies (1 yoga, 2 theater, and 1 dance) reported non-significant effects, with majority of the remaining studies suggesting small to medium-sized improvements. From the studies that reported training-related affective improvements, we were able to calculate a total of 57 ES, with 5 ES calculated from 4 out of the total 5 level I studies, 34 ES obtained from 3 out of the total 6 level II studies, and 18 ES from 4 out of the total 9 level III studies assessing changes in affective states following CMT.

Among the 5 Level I studies that indicated positive effects following CMT (1 music, 1 dance, 3 theater), largest ES estimates were reported by the 2 studies by Corbett et al. that found improvements in affect/emotion recognition and a reduction in anxiety (ES: 0.49–0.97) following a 10-week Social Emotional NeuroScience Endocrinology (SENSE) theater intervention in 8–14-year-old children with ASD (87, 165). While the Level I dance study by Koehne et al. (171) found significant improvements in emotion interference and empathy (ES: 0.31) on a computerized Multifaceted Empathy Test (MET) following a 10-week imitation- and synchronization-based group DMT in youth and adults with ASD, the single Level I music-based study by Srinivasan et al. (118) reported only within-group improvements (ES: 0.32–0.43) in levels of negative and interested affect in the group receiving rhythm therapy.

Of the 6 Level II studies (1 music, 1 yoga, 1 theater, 2 dance, and 1 combined music and dance), a majority of the studies reported small to medium positive effects on empathy, emotional synchronicity, joy, and overall psychological well-being inclusive of anxiety, depressed affect, tension, and vitality (ES: 0.31–0.68). For example, Kim et al. used a within-subject comparison cross-over design for improvisational music therapy and toy play sessions in 10 children with ASD and found that children showed greater frequencies of joyful events and mirrored emotional synchronicity (ES: 0.47–0.55) with the therapist during music therapy sessions compared to toy play sessions (101). The 9 Level III pre-post designs provided the largest variations in ES estimates ranging from small to large (0.3–1.12) across multiple studies for multiple outcomes related to anxiety, self-esteem, empathy, resiliency, emotion recognition, and enjoyment during sessions. However, out of the total 57 calculated ES across all affective outcomes from 11 studies, only 3 ES—from one Level III music and one Level I theater-based intervention (~18% studies) had a CI that did not include 0. Moreover, similar to social communication outcomes, although a majority of theater-based studies assessed affective outcomes, our calculations suggest that only 1 study found significant, non-zero effects of the intervention on affective outcomes. Overall, our review suggests that although individual studies concluded *small-to-large-*sized positive effects, there is at present *insufficient evidence* supporting the beneficial effects of CMT on *affective* outcomes in ASD.

Taken altogether, we found only limited evidence from 26% studies (8 out of 31 studies) that were mainly Level II and Level III studies with high risk-of-bias for beneficial effects of yoga, music, martial arts, theater, and dance on behavioral-affective outcomes in ASD (see **Table 6**).

#### Sensorimotor

Of the three studies that assessed *sensory* skills using either questionnaires (Short sensory Profile, Questionnaire of movement therapy) or video coding-based measures, 2 Level II studies reported moderate-sized positive effects following dance and music interventions on children's body awareness and their negative response to touch (77, 130), with the 3rd theater-based study (Level III) reporting non-significant effects (124). We could calculate only 2 ES from 2 out of 2 Level II studies of which only 1 ES from a Level II music study did not include 0. Specifically, an 8-week intervention of music-based movement therapy led to improvements in children's negative response to touch (ES: 0.59) during training sessions compared to a waitlist control group (130). Although Koch et al., reported moderate improvements in awareness of body movement (ES: 0.62) after a 7-week long manualized DMT intervention, the CI of the calculated ES included 0 [(77); see Tables 4A–C]. Given the few studies that have assessed effects of CMT on sensory outcomes, at present, there is *insufficient evidence* to make definitive conclusions on the effects of CMT approaches on *sensory* outcomes in ASD.

Fifteen studies that assessed *motor* outcomes, i.e., 5 Level I (1 music, 2 yoga, 2 martial arts), 6 Level II studies (2 music, 1 yoga, 1 martial arts, 1 dance, 1 music and dance combined), and 4 Level III studies (2 music, 1 yoga, 1 martial arts) used standardized tests such as the Bruininks-Oseretsky Test of Motor Performance-2nd Edition (BOT-2), Movement Assessment Battery-2nd Edition (MABC), and the Korperkoordinationstest fur Kinder (KTK), questionnaires such as the ATEC and imitation test battery, as well as observation-based quantitative measures such as posturography, and video-coding for imitation and interpersonal synchrony. Out of the 8 studies that reported ES estimates, five studies (1 music, 1 yoga, 2 martial arts, 1 music and dance combined) reported medium-to-large positive effects of CMT and 3 music-based studies (1 Level I, 1 Level II, and 1 Level III; see Tables 2A,B) reported non-significant effects on motor skills following intervention (120, 138, 140). However, even among the clinical trials, 3 studies (27, 150, 157) reported only within-group effects suggesting that the positive effects were perhaps not robust enough to attain statistical significance at the between-group level. We were able to calculate a total of 18 ES, i.e., 10 ES from 4 out of the total 5 Level I studies, 5 ES from 1 out of the total 6 Level II studies and 3 ES from 2 out of the total 4 Level III studies assessing motor outcomes.

Large effects were obtained from the Level I study by Sarabzadeh et al. (158) on ball skills and balance subscales (ES: 3.14–3.16) assessed using the standardized MABC-2 test after a 6-week Tai Chi Chuan martial arts-based intervention. Similarly, the single Level II dance intervention that assessed motor outcomes reported improvements in the Korperkoordinations test fur Kinder (KTK) test (ES: 0.88–2.63), a measure of neuromuscular coordination including balance and agility, following an 8-week traditional Greek dance program in 5 children with ASD (85). Of the 3 studies that employed quantitative measures to assess motor outcomes, Garcia et al. in a Level III study reported large improvements in moderate-to-vigorous physical activity levels (MVPA) (ES: 0.97) using Actigraph GT9X accelerometers following a judo intervention in children with ASD (127). The other Level I and II studies reported improved static and dynamic balance and reduced postural sway during an eyes closed single leg balance task, respectively (ES: 0.5–5.34), following a 10-week Kata and an 8-week Taekwondo intervention in children with ASD, respectively (157, 159). Overall, out of the 18 ES calculated from 7 studies, CI for 8 ES calculated from 4 Level I studies (2 yoga, 2 martial arts) and 1 Level II study (music) did not include 0 (~71% studies). Therefore, there seems to be *limited, yet very promising* evidence from mainly Level I studies for *medium-to-large sized improvements* in *motor* outcomes following martial arts and yoga-based interventions in ASD.

Therefore, altogether across the sensorimotor domain, there is limited evidence from around 28% studies (5 out of 18 studies, i.e., 4 Level I and 1 Level II) which showed improvements in assessed outcomes following predominantly yoga and martial arts interventions (see **Table 6**).

#### Cognitive

The 6 Level I studies (2 yoga, 4 martial arts) that assessed cognition used EEG measures to record neural activity, computerized tests such as the Go-No-Go and the Hearts and flowers test, as well as questionnaires such as the ATEC to report medium to large ES for improvements in executive functioning, visual memory, cognitive awareness, and brain activation patterns following CMT (104, 148, 152, 154, 155, 161) (see Tables 3A,B, **6**). For instance, Chan et al. reported large improvements in self-control (ES: 0.84), indicated by a reduction in the # of rule violations during a Tower of London task following a Nei Yang Gong martial arts intervention compared to a control group that received progressive muscle relaxation (154). Similarly, following a 13-week mixed martial arts intervention, Phung and Goldberg reported improvements in accuracy (ES: 0.83–1.01) on the computerized Hearts and Flowers executive functioning test (104). Of the 22 ES we could calculate from the 6 studies, the CI of 14 ES from 4 Level I studies (2 yoga, 2 martial arts) did not include zero (~66% studies) (see **Table 6**). We found some disagreements between reported and calculated ES (see Tables 4A–C for details) (104, 154); however, our overall assessment suggests *limited promising evidence* for *medium-to-large sized improvements* in *cognitive* skills following martial arts and yoga-based interventions.

#### Functional Skills and Quality of Life

Three studies assessed activities of daily living and QOL using self- and family-report questionnaires such as the World Health Organization-Five Well-being Index (WHO-5), the WHO Disability Assessment Scale (WHODAS) and the Functional Independence Measure (FIM). Of these, 1 study each of Level I and II, respectively (1 music and 1 dance + EAT) [(116, 174); see Tables 3A–C, **6**] reported non-significant between-group effects on QOL and functional participation, whereas a single Level III study found medium-sized within-group improvements in QOL following a yoga-based intervention (146). Specifically, de Bruin et al., reported medium-sized improvements in QOL in adolescents with ASD measured on the WHO-5 well-being index (ES: 0.55–0.63) following a 9-week mindfulness training intervention (146). Although Souza-Santos et al., reported non-significant between-group differences, they found within-group improvements in the dance and combined dance and equine-assisted therapy intervention groups on the Functional Independence Measure (FIM) and WHO Disability Assessment Scale (WHODAS) (174). Based on reported data, we could only calculate 6 within-group ES from a single Level II study. Our calculations confirmed the findings from Souza-Santos et al. (174) with within-group ES estimates ranging from 0.64 to 0.73 and their CI not inclusive of 0. However, overall, at present, there is *insufficient evidence* to indicate any benefits from CMT on *functional participation and QOL* of individuals with ASD.

#### Other Domains

Sixteen (4 Level I, 6 Level II, 6 Level III) out of the 72 papers assessed effects of CMT on other domains including, (1) physiological parameters such as sleep, gastrointestinal (GI) problems, heart rate variability, and cortisol levels, (2) training-specific skills such as musical abilities and mindful awareness, and (3) cost effectiveness of provided interventions and parent-reported adverse effects (see Tables 3A–C, **6**). A combination of quantitative measures such as ECG recordings and salivary cortisol levels, standardized tests, and patient/caregiver-report questionnaires were used to assess these miscellaneous outcomes. For instance, Corbett et al. assessed the effects of theater-based interventions on salivary cortisol levels, a marker for physiological stress, in 3 separate studies, 2 of which were Level III pre-post designs and one was a Level I RCT (87, 124, 126). While theater interventions had large within-group effects (ES: 0.73–2.55) for reducing cortisol levels in participants during and after the intervention (124, 126), the effects were not strong enough to attain significance at the between-group level (87). A single Level II study also reported significant medium-sized effects (ES: 0.3–0.64) on a questionnaire-based assessment of sleep and GI problems in children with ASD following a 90-day yoga training program (147). Two Level III studies that assessed training-specific musical skills reported mostly large effects (ES: 1.13–2.67) on musical vocal behaviors, rhythmic imitation of musical patterns, turn taking within musical contexts, instrument playing, and singing following music therapy sessions (129, 131). No statistically significant effects were demonstrated on mindful awareness and heart rate variability following CMT (151). Three studies reported positive trends in qualitative data on parent/teacher and participant satisfaction, feasibility of implementation, and social validity of CMT (116, 140, 166).

We were able to calculate a total of 66 ES from 7 studies, i.e., 9 ES from 2 out of the total 4 Level I studies, 31 ES from 1 out of the total 5 Level II studies and 26 ES from 4 out of the total 6 Level III studies. Out of these 66 calculated ES, 8 ES from 2 Level I studies (1 yoga, 1 theater) and 12 ES from 4 Level III studies (2 music, 2 theater) suggested mostly medium-to-large-sized effects on assessed outcomes (see Tables 4A–C, **6**). Overall, there is *preliminary evidence* from mostly within-group designs supporting the effectiveness of music therapies in enhancing children's musical skills and theater-based interventions in improving salivary cortisol levels and reducing stress in individuals with ASD.

### Short- and Long-Term Effects of CMT

The efficacy and utility of any therapy depends not only on the effects assessed during and immediately following the intervention, but more importantly on the carryover of training effects into real-world settings beyond the duration of the training. Only 17 (7 Level I, 7 Level II, 3 Level III studies) out of the 72 studies assessed the short- and long-term effects of CMT through follow-up (FU) testing that was conducted between 2 weeks and 12 months post-intervention. Of the 17 papers, only 9 (2 music, 1 yoga, 3 martial arts, 3 theater) studies found sustained improvements in outcomes at FU. The music studies (116, 140) that compared rock drumming and improvisational music therapy, respectively, with standardized care found improvements in social and motor outcomes at 2 weeks and 12 months FU, respectively (see Table 3A). Three martial arts-based studies from a single group of authors found retention of positive improvements on behavioral and social communication outcomes at 1-month FU (see Table 3B) (86, 122, 153). Sustained improvements were also seen following the SENSE theater and SDARI interventions in social communication and behavioral domains (see Table 3C) at 1.5- and 2-months post-intervention respectively (163, 165). The only study in the review that conducted multiple FU sessions (at 3, 6, and 12 months) assessed the effects of a drama-based intervention on social communication and behavioral outcomes (170). However, the study only reported outcomes at the final FU visit and suggested sustained improvements in autism severity and emotion recognition at 12 months post-intervention compared to baseline values (170). Lastly, 1 yoga-based intervention study found sustained improvements in executive functioning at 1.5 months post intervention compared to the posttest and baseline measures (152). On the other hand, 8 studies (3 music, 1 yoga, 1, martial arts, 1 theater, 2 dance) found that the immediate beneficial effects of CMT were not sustained at FU (87, 134, 136, 137, 146, 172, 173). Overall, although 9 of the 17 papers claimed sustained beneficial effects of CMT in individuals with ASD, our calculations suggested that only 4 out of the 22 calculated ES (ES: −0.79–0.71) from a Level II yoga and a Level III theater-based intervention study (152, 170) were statistically significant (i.e., CI did not include 0). Thus, there is currently *insufficient evidence* for *short-to-long-term sustained benefits* following CMT in individuals with ASD.

## Discussion

### Summary of Results

Creative movement therapies have been an ongoing topic of study over the past 3 decades. Within CMT approaches, the effects of music- and yoga-based therapies have been studied since the 1990s and 2000s, whereas dance, theater, and martial arts have been studied only more recently over the past decade. Given that this area of study is still in its infancy, there is presently lack of rigorous, definitive evidence supporting the use of CMT approaches within the standard-of-care clinical practice in ASD. There have been a few reviews in the past assessing the individual effectiveness of music, yoga, martial arts, theater, and dance approaches in individuals with ASD. Despite the common underlying theoretical framework and the key intervention ingredients across these different approaches, to date, there has been no umbrella review that has systematically compiled evidence across different types of CMT approaches in individuals with ASD. Our paper addresses this critical gap by providing a comprehensive review of the literature through August 2021, supplemented with a critical risk of bias assessment on different CMT approaches as applied to individuals with ASD. By conducting both a narrative literature synthesis and a quantitative review through calculation of ES estimates of treatment effects, we are able to systematically compare and contrast the efficacy of different types of CMT approaches in individuals with ASD.

Of the total 72 studies, we were able to calculate within- and/or between-group ES estimates for around 89% studies (23 music, 7 yoga, 16 martial arts, 10 theater, and 7 dance, 1 music and dance) of which around 45% studies (*N* = 29) showed statistically significant, non-zero effects of CMT on assessed outcomes across domains. Specifically, we found evidence for (1) medium- to large-sized improvements in social communication skills from over 30% of the studies (mostly Levels I and II) that assessed these outcomes, (2) medium-sized improvements in the behavioral domain from around 33% studies (mostly Levels II and III) that assessed these skills, (3) medium-to-large improvements in motor outcomes from around 33% studies (mostly Level 1) that assessed movement performance, and (4) medium-to-large improvements in cognitive skills from over 65% (all Level I) of the studies that assessed this domain. In comparison, we found limited evidence to date for the positive impact of CMT on sensory, affective, and functional participation domains. In terms of CMT types, our review suggests that there is presently strongest evidence for the beneficial effects of music-based therapies in promoting social communication skills (5 out of 7 Level I music studies), followed by limited, yet positive evidence for both martial arts and yoga in promoting motor and cognitive skills (2 Level I studies for each approach for each domain), and for martial arts in promoting social communication skills (2 out of 3 Level I studies). Below we summarize the potential mechanisms of change for individual CMT approaches.

### Music Therapy Interventions

Our literature search revealed the largest number of studies for music-based interventions compared to all other CMT approaches, with around 40% studies reporting significant improvements (ES: 0.02–4.11) in measured outcomes. Specifically, 35% studies (*N* = 7) showed improvements of varying sizes in social communication skills (ES: 0.02–4.06) and around 38% (*N* = 3) studies suggested large improvements in behavioral-affective outcomes (ES: 1.28–4.11).

Previous literature in the field of music and autism suggests that children with ASD particularly enjoy musical experiences, and in fact have enhanced musical perception skills (53, 178). From a brain imaging standpoint, there is substantial evidence that musical practice promotes multimodal integration by activating long range connections that simultaneously engage the auditory, visual, somatosensory, motor, and premotor areas as well as brain networks such as the mirror neuron system that are especially dysfunctional in ASD (52, 101, 106). Given the considerable overlap between brain substrates underlying speech and music, and the overall structural similarity between music and language, it has also been argued that musical training can in fact lead to enhanced speech processing in individuals with ASD (52). Overall, there is considerable behavioral and neuroimaging evidence to support the mechanisms for beneficial effects of music-based training in ASD (179).

Our review findings of consistent improvements in social communication skills following music therapies is not surprising given that such activities are based in rhythm, melody, and harmony, and involve components of singing, listening, music making with instruments, moving to the beat of the music, and spontaneous improvisation, all of which provide abundant opportunities for practice of social communication skills such as turn taking, joint attention, imitation, and verbal communication (53). Several of the reviewed studies also provided opportunities for flexibility and child-led activities during training that probably fostered children's engagement and led to better outcomes (4, 6, 27, 118, 119, 135). Although authors also hypothesized that musical training provides a non-intimidating context that may contribute to a reduction in off-task behaviors, stereotypes, and other repetitive behaviors in participants (130, 131), there is presently a need for more rigorous, high-quality research to support the use of music-based approaches in improving behavioral impairments in ASD. Moreover, music making using different types of instruments challenges the fine motor and cognitive systems as it typically involves complex, sequential, and precise finger and hand movements that require intricate motor planning and execution (53). Although the current evidence on the effects of music-based interventions on sensorimotor and cognitive skills is scant, this is definitely an area that deserves further attention.

Taken altogether, although music therapy approaches have the strongest evidence among other CMT approaches, there is a clear need for more research to assess the multisystem effects of these interventions on primary and secondary comorbidities in ASD.

### Yoga Therapy Interventions

Yoga-based interventions fall under the category of holistic mind-body therapies and are based on principles and techniques of yogic practice that date back several millennia to ancient India. Yoga and mindfulness practice has been postulated to have physical, mental, and spiritual effects (180, 181). Studies included in our review evaluated the effects of yoga practice on behavioral, social communication, and motor skills as well as on physiological parameters, with very few studies assessing effects on affective control, cognitive, and functional skills. We were able to calculate ES estimates from around 55% of all yoga studies; nevertheless, a majority of the studies indicated medium-to-large improvements (ES: 0.43–2.66) in measured outcomes. Our analyses indicated promising beneficial effects in behavioral regulation (*N* = 3, ES: 0.43–2.66), motor (*N* = 2, ES: 0.76–2.66), and cognitive skills (*N* = 2, ES: 0.91–1.42) following yoga-based interventions.

Yoga-based programs emphasize the practice of breathing control and mindful body awareness that may help individuals with ASD manage their behavioral and mood/affective symptoms (88, 145, 148, 150). Moreover, the practice of static and dynamic postures that focus on improving balance, core muscle strength, flexibility, and body awareness may impact the sensorimotor systems (150) and also have physiological effects on digestion, sleep, and HR variability (88, 145, 148); see Table 5 for components). Yoga and mindful awareness have also been found to help with attention regulation, memory, and executive control (152, 154, 155). Additionally, the only study that provided mindfulness training to adolescents with ASD and their caregivers found improved quality of parent-child interactions as evidenced by decreased parental stress and improved behavioral regulation (146). Despite extensive evidence for the whole-body effects of yoga in healthy individuals (182–184), our review suggests that the use of yoga-based interventions in ASD is presently an under-researched topic that deserves greater systematic investigation.

**TABLE 5:** Intervention guidelines for CMT interventions based on reviewed literature.

**Characteristics**	**Suggested guidelines for clinicians**
**Music**	
Duration	8–16 weeks
Frequency	1–2 times per week
Time	1–1.5 h per session
Type	- Improvisational music therapy - Relational therapy - Family-centered music therapy
Components	- Hello songs and whole body warm-up activities - Music making using instruments like shakers, drums, tambourines, cymbals, maracas, etc. - Music making allowing child opportunity to explore instruments and music - Gross motor movements to the beat of music. Could involve turn taking or imitation-based rhythmic synchronization games where children match up movements to tempo of music and to actions of social partners - Cool-down and farewell songs
**Yoga**	
Duration	8–16 weeks
Frequency	3–5 times per week
Time per	0.5–1 h per session
Type	- Relaxation - Creative yoga - Mindfulness training - Mandala yoga
Components	- Whole body warm-up activities - Individual poses with holds, focusing on upper body and lower body flexibility, core strength, and balance - Partner poses involving joint yoga poses with a social partner - Mindfulness practice and breathing exercises - Whole body relaxation
**Martial arts**	
Duration	12–14 weeks
Frequency	2–4 times per week
Time	1 h per session
Type	Kata, Karate, Tai Chi, Mixed Martial Arts, Judo, Taekwondo, and Mind-body exercise
Components	- Whole body warm-up activities - Practice of individual movements/postures depending on type of martial art for example, punches and kicks for Kata or Judo, gentle poses and stretches for Tai Chi, etc. - Practice of sequences/flows that involve multiple movements put together - Cool down stretches
**Theater**	
Duration	10–12 weeks
Frequency	3–5 times per week
Time	2–4 h per session
Type	SENSE theater, SDARI, SCIP
Components	- Warm up games and theatrical improvisational games - Dramatic activities focused on facial expressions, body language, emotions, perspective taking, group cohesion, voice modulation, verbal and non-verbal communication, and acting
	- Script reading, character development, script memorization- Role playing and dialogues - Rehearsals of scenes and theatrical performances - Peer-mediated theater activities - Theatrical games, role-play and rehearsals - Improvisational activities targeting imitation, modeling, etc. - Public performances on completion of training
**Dance**	
Duration	12 weeks
Frequency	2 times a week
Time	1 h per session
Type	DMT
Components	- Whole body warm-up activities - Individual activities focused on exploring body movement flow in space and on increasing body awareness with and without music - Interactive imitation, mirroring, and synchronization-based choreographed activities in dyads, small groups, and larger groups - Improvisational dance sequences - Verbal reflections - Cool down and relaxation
**General recommendations**	
Setting	- Indoor settings like the child's home, child's school, community center, etc. - Outdoor open settings that provide space for free movement
Intervention providers	- Specialized instructor (music therapist, yoga certified teacher, dance movement therapist, etc.), - Licensed professionals (PT, OT, SLT)
Assistants	- Caregivers, school staff, undergraduate students or research assistants

### Martial-Arts Interventions

Around 56% of reviewed martial arts-based studies reported significant small-to-large improvements in measured outcomes (ES: 0.29–5.34), specifically in social communication (*N* = 4, ES: 1.13–1.15), cognitive (*N* = 2, ES: 0.42–1.19), and motor (*N* = 2; ES: 3.14–5.34) domains. Moreover, improvements in the cognitive domain i.e., in executive functioning, which includes working memory, flexible thinking, and inhibitory control are supported by high-quality Level I studies [(104, 154, 161); see Table 6]. This is not surprising given the heavy emphasis in martial arts training on discipline, structured practice of multistep action sequences, and movement precision, all of which require focused attention, motor planning, task switching, and working memory (75, 104, 123, 154, 159).

**TABLE 6:** Summary of reviewed studies that assessed specific domains and number of studies that showed improvements based on ES calculations.

**CMT approach**	**Social communication** **(*N* = 47)**	**Behavior** **(*N* = 21)**	**Affective** **(*N* = 20)**	**Sensory** **(*N* = 3)**	**Motor** **(*N* = 15)**	**Cognitive** **(*N* = 6)**	**Functional participation** **(*N* = 3)**	**Other** **domains** **(*N* = 16)**
Music	20 (7)	5 (2)	5 (1)	1 (1)	5 (1)	0 (0)	1 (0)	5 (2)
Yoga	4 (1)	4 (3)	3 (0)	0 (0)	4 (2)	2 (2)	1 (0)	4 (1)
Martial arts	7 (4)	3 (1)	0 (0)	0 (0)	4 (2)	4 (2)	0 (0)	2 (0)
Theater	12 (3)	6 (1)	8 (1)	1 (0)	0 (0)	0 (0)	0 (0)	5 (3)
Dance and combined approaches	4 (0)	3 (0)	4 (0)	1 (0)	2 (0)	0 (0)	1 (0)	0 (0)

*Number mentioned outside parenthesis indicates number of studies that assessed outcomes related to specific domains and number within parenthesis indicates the number of studies that showed improvements in specific outcomes based on our ES calculations*.

The reviewed studies also suggested the potential for martial arts training to impact socialization, behavior, and motor skills. For instance, martial art training led to improved synthesis and metabolism of neurotransmitters oxytocin, serotonin, and dopamine (75, 122). In fact, disturbed metabolism in these very neurotransmitter systems is thought to underlie the social dysfunction and stereotypies commonly seen in individuals with ASD (75, 122, 153). Similarly, high-energy, dynamic, martial art movement routines are thought to physically resemble stereotypical movements characteristic in ASD, perhaps serving as a functional “substitute” for repetitive behaviors, while still providing the same level of sensory input and reinforcement (75, 122). Although there is presently evidence from only 2 Level I studies, it seems plausible that martial arts training may also have effects on the sensorimotor system through practice of poses and action sequences that require good postural control, balance, multi-limb coordination, strength, agility, and optimal processing in the vestibular- and tactile-proprioceptive systems (104, 158). Our review of the existing literature suggests that among all CMT approaches, martial arts-based therapies seem to have the strongest evidence at present for promoting multisystem development in social communication, behavior, motor, and cognitive domains.

### Theater-Based Interventions

Although a majority of theater-based studies assessed social communication and behavioral-affective outcomes, only around 42% (i.e., 2 Level I, 1 Level 1 and 2 Level III studies) of reviewed studies reported medium-to-large improvements (ES: 0.56–2.55) across these domains as well as a reduction in cortisol levels (ES: 0.73–2.55) following theater training. In fact, 2 of these studies were conducted by the same research group (126, 165). The reviewed theater studies typically provided training in a group format emphasizing interactions with peer models, specialized instructors, teachers, and other staff (87). Such a socially embedded and interactive context may provide plenty of opportunities for individuals with ASD to practice critical social communication skills such as joint attention, turn taking, perspective taking, and dialogue delivery, while also learning to recognize and express subtle socio-emotional cues related to facial expressions, voice intonation, and body language. It is therefore surprising to see a lack of statistically significant effects in support of enhanced social communication and behavioral-affective skills following theater training. A salient difference between theater and other CMT approaches is the average session duration, with theater interventions on an average lasting for much longer, i.e., around 2.9 h/session (see Table 5). It remains to be seen if the long duration of intervention sessions impacted abilities of individuals with ASD to sustain engagement during the training program. Overall, despite the highly interactive nature of theater, at present, there is insufficient evidence to support the use of theater-based training to facilitate social communication and behavioral-affective skills in individuals with ASD.

### Dance Therapy and Combined Interventions

Although all individual reports (*N* = 8; 2 Level I, 6 Level II) concluded positive effects of dance-based therapies, our ES calculations from data reported in 6 papers suggested that none of the ES were statistically significant. This was the singular approach where studies recruited participants across the lifespan from 14 to 65 years. The wide age-range might have added to the variability of data, undermining the effects reported in these studies. Dance is an embodied experience incorporating elements of complex coordination, motor planning, and balance that may provide individuals with ASD opportunities to express their emotions through fluid bodily movements and to engage in mirrored practice during group choreography (172, 173). Despite the potential for promising effects on multiple systems through the very embodied nature of the experience, the current evidence on dance therapy in ASD is very limited. We call for future research to fully explore the use of dance-based interventions in individuals with ASD. We specifically recommend that future studies assess the effects of dance approaches on participants within a narrower age range.

### Implications for Clinical Practice

Based on the studies reviewed, we suggest intervention guidelines for clinicians working with individuals with ASD in terms of assessments and interventions pertaining to CMT approaches. In terms of assessment measures, we recommend that clinicians use a combination of domain-specific standardized tests, observational measures, parent report questionnaires/interviews, and video coding to assess the impact of CMT approaches on multiple systems (see Tables 3A–C). Ultimately, the choice of assessment tools will depend upon multiple factors including the domains assessed as well as participant characteristics such as age, autism severity, functional level, receptive and expressive communication, and intellectual abilities. From our own experience, we recommend that objective clinician-based assessment tools be supplemented with parent reports to allow assessment of the individual's skills across a variety of structured and naturalistic activities and environments. We also recommend that researchers collect video data of testing and training sessions that can be scored at a later time by unbiased coders, thereby again allowing an evaluation of multiple snapshots of target behaviors across a variety of settings such as the lab, home, school, etc.

In terms of treatment, Table 5 provides a summary of our suggested intervention guidelines for the different CMT training approaches in terms of FITT principles (*F*requency, *I*ntensity, *T*ime, *T*ype). Note that our guidelines are based on the reviewed literature, specifically, training programs that led to appreciable improvements in assessed treatment outcomes. The choice of CMT approach should ultimately depend on the preferences of the individual with ASD/their family. Clinicians should choose the approach that their client is most excited about and that they are comfortable delivering. Moreover, based on the evidence from this review, we recommend that there is at present, most consistent evidence from high-quality studies with low risk of bias for enhancements in social communication skills following music and martial arts interventions, and in motor and cognitive skills following yoga and martial arts practice. There is need for more systematic research to support the use of theater and dance-based approaches in the plan of care of individuals with ASD.

To administer CMT interventions to their clients with ASD, allied health professionals may need to consult with certified instructors and work collaboratively with them to tailor interventions to their client/family's needs. Moreover, several studies reported using common training strategies derived from conventional ASD treatments such as ABA, PECS, TEACHH, etc. while delivering CMT approaches with individuals with ASD. While structured practice will be an integral part of every CMT-based session, we strongly recommend that clinicians reserve time during sessions for free play and improvisation that will afford individuals with ASD opportunities for creative movement exploration and self-expression. Although there is a need for more rigorous research in this field, our review certainly suggests that CMT approaches involve embodied experiences that engage multiple systems/domains, are fun and engaging, and may provide individuals with ASD a variety of activity options fostering lifelong learning and creative expression.

### Implications for Research

Around 75% of the reviewed studies employed between-group designs; however, <50% of the total studies were RCTs which are considered the gold standard for intervention efficacy research. There is a need for greater methodological rigor of clinical trials to reduce risk of bias by ensuring random and concealed assignment of participants to intervention and control groups, blinding of therapists and assessors, ensuring baseline similarity between groups prior to group assignment, and employing intention-to-treat analyses when possible. Since RCTs require significant amount of financial and personnel-related resources and are not always feasible to conduct in clinical settings, several studies in our review used pre-post designs. Our risk of bias assessment for pre-post designs indicated a need for better justification of sample sizes using power analyses, administration of tests at multiple times to obtain stable estimates of the child's behavior at baseline and post-intervention, better assessment and reporting of validity and reliability of selected outcome measures, and blinding of assessors to ensure unbiased estimates of participant performance.

Overall, across all study designs, we recommend that future studies provide more information on sample characteristics within the original report including measures of autism severity, IQ levels, as well as functional skills assessed using parent questionnaires such as the VABS. This is crucial since, the effects of CMT approaches might differ across participants based on these above-mentioned characteristics. Interestingly, a very small proportion of the reviewed studies included participants with intellectual disability and similarly even fewer studies recruited youth and adults with ASD, suggesting a need for more research with these subject demographics. In terms of study quality, future studies should report on steps taken to assess and ensure treatment fidelity during intervention delivery.

Among reviewed CMT approaches, the greatest quantity of evidence is for music-based interventions; there is therefore, a need for more rigorous research on other CMT approaches as well as efforts directed toward replication of the effects of music-based therapies on multiple systems in ASD using large sample size studies. Specifically, our review suggests that yoga- and martial arts-based therapies may be promising to promote multisystem development in individuals with ASD. Although reviewed studies assessed a variety of outcomes, the most frequently assessed domains included social communication and behavioral skills. Given the embodied nature of CMT approaches and their proposed mechanism of action on multiple systems, it would be important for future studies to holistically assess other developmental domains including sensorimotor, affective, and cognitive systems that also present as significant challenges for individuals with ASD. Moreover, researchers should go beyond the impairment domain and begin assessing the impact of CMT approaches on function and participation of individuals with ASD. In order to understand the carryover effects of CMT approaches, studies will need to assess treatment effects both in terms of short-term effects i.e., immediately following intervention completion, as well as the long term maintenance of training-related gains at follow-up. Finally, we urge that authors include their data within the original reports to enable calculation of ES estimates for measured outcomes and meta-analytic analyses.

### Limitations

Although we used a comprehensive search strategy (see Appendix 1) to identify eligible studies, it is possible that we may have missed relevant research. For the purposes of our review, we only included clinical trials and pre-post study designs with the exclusion of case studies, narrative reports, and other types of qualitative reports. We also excluded conference proceedings and unpublished theses and dissertations from this review. Finally, we limited our review to only articles published in English. Although our original intention was to restrict our review to studies that used CMT approaches in individuals with ASD, there were a few papers (*N* = 5) in our review that recruited mixed samples, thereby adding to the heterogeneity of the sample. In terms of intervention components, several studies reported using training strategies derived from conventional, evidence based ASD treatments while delivering CMT to participants. Therefore, at present, the literature does not allow us to tease apart the true effects of the key ingredients of the CMT approaches themselves vs. those of the training strategies used in conjunction with the CMT approaches. In a related vein, a majority of the studies did not report on acceptability, implementation feasibility, cost-effectiveness, etc. of interventions. The extent of the literature does not presently allow the development of clinical practice guidelines for the ASD population; instead, the suggested treatment guidelines reported in our paper are based only on the reviewed studies and the dosing parameters that led to gains in measured outcomes in the reviewed studies.

## Conclusions

Our systematic review aimed at providing a comprehensive summary of the literature through August 2021 on the effects of various types of CMT approaches for individuals with ASD. Our search identified a total of 72 articles that used music, yoga, martial arts, theater, and dance-based intervention programs in 1,939 number of individuals with ASD between 3 and 65 years. Our quantitative synthesis of the published literature suggested strong and consistent evidence for small-to-large improvements in social communication skills following music and martial arts training as well as medium-to-large sized improvements in motor and cognitive skills following martial arts and yoga training. Presently, there is limited evidence in support of theater and dance-based approaches as well as the utility of all CMT approaches in improving affective, sensorimotor, and functional participation skills in individuals with ASD. Our review offers future directions for research examining the effects of CMT approaches in ASD as well as provides intervention guidelines for clinicians to incorporate CMT approaches in their plan of care for their clients with ASD.

## Data Availability Statement

The original contributions presented in the study are included in the article/Supplementary Material, further inquiries can be directed to the corresponding author.

## Author Contributions

SS was responsible for conception of the manuscript. WCS, AB, and SS were involved in project design. NA and SS finalized and coded the articles and were responsible for data extraction and analyses. NA and SS put together the first draft of the manuscript. All authors were involved in proof reading and editing of the manuscript prior to submission.

## Funding

AB's research was supported by a Clinical Neuroscience Award from the Dana Foundation and multiple grants from the National Institutes of Health (Grant #s: 1S10OD021534-01, P20 GM103446, 1R01-MH125823-01). SS's efforts on this project were supported through multiple internal grants from the University of Connecticut: Scholarship Facilitation Fund (SFF), Research Excellence Program (REP) Award, and a seed grant from the Institute for Collaboration on Health, Intervention, and Policy (InCHIP).

## Conflict of Interest

The authors declare that the research was conducted in the absence of any commercial or financial relationships that could be construed as a potential conflict of interest.

## Publisher's Note

All claims expressed in this article are solely those of the authors and do not necessarily represent those of their affiliated organizations, or those of the publisher, the editors and the reviewers. Any product that may be evaluated in this article, or claim that may be made by its manufacturer, is not guaranteed or endorsed by the publisher.
