# Supplementary material for: Effects of Creative Movement Therapies on Social Communication, Behavioral-Affective, Sensorimotor, Cognitive, and Functional Participation Skills of Individuals With Autism Spectrum Disorder: A Systematic Review
Source: Front Psychiatry. 2021 Nov 18;12:722874. doi: 10.3389/fpsyt.2021.722874 (PMC8637167; doi:10.3389/fpsyt.2021.722874)
Supplement: Supplementary file 1 [file Data_Sheet_1.docx]

**Appendix**

*Appendix 1: Search terms for data bases*

**^^[[1]](#footnote-1)^^PubMed:**

(“play therapy”[mesh] OR "Sensory Art Therapies"[Mesh:NoExp] OR "Play and Playthings"[Mesh:NoExp] OR "Games, Recreational"[Mesh] OR "Art Therapy"[Mesh] OR "Dance Therapy"[Mesh] OR "Music Therapy"[Mesh] OR “therapeutic play” OR “filial therapy” OR fun OR “play”[ti] OR “play behavior” OR “play behaviors” OR “play behaviour” OR “group play” OR playful* OR “play therapy” OR “play therapies” OR “play ground” OR playground* OR (“imaginary” AND “play”) OR “imaginative play” OR “social play” OR “tumble play” OR “creative play” OR “physical play” OR “symbolic play” OR “fantasy play” OR “outdoor play” OR “exploratory play” OR “object play” OR “role play” OR “playing” OR playtime OR “play time” OR playground* OR “plaything” OR “playthings” OR “game” OR “games”[tiab] OR “toy” OR “toys” OR puppet* OR “building blocks” OR craft[tiab] OR crafts OR scissor* OR crayon* OR Lego OR legos OR “Play-doh” OR Duplo OR coloring OR (drawing AND paper) OR “music”[tiab] OR “musical” OR art[tiab] OR arts[tiab] OR artistic OR dance[tiab] OR dances OR dancing OR recess OR gymnastic* OR running[mesh] OR running[ti] OR bicycling[mesh] OR bicycle* OR cycling[ti] OR tricycle* OR yoga OR pretend*)

AND "Asperger Syndrome"[Mesh] OR "Autism Spectrum Disorder"[Mesh] OR autism[tiab] OR autistic* OR Asperger*)

AND ("randomized controlled trial"[pt] OR "controlled clinical trial"[pt] OR "clinical trials as topic"[mesh] OR "random allocation"[mesh] OR "double-blind method"[mesh] OR "single-blind method"[mesh] OR "clinical trial"[pt] OR "research design"[mesh:noexp] OR "comparative study"[pt] OR "evaluation studies"[pt] OR "follow-up studies"[mesh] OR "prospective studies"[mesh] OR "cross-over studies"[mesh] OR "clinical trial"[tw] OR ((singl*[tw] OR doubl*[tw] OR trebl*[tw]) AND (mask*[tw] OR blind*[tw])) OR placebo*[tw] OR random*[tw] OR "control"[tw] OR "controls"[tw] OR prospectiv*[tw] OR volunteer*[tw])

NOT (“game theory” OR “gaming” OR video[ti] OR “computer game” OR “computer games” OR Nintendo OR “wii”[ti] OR gambling OR “state of the art” OR musician*[ti] OR “assisted reproductive technology” OR “internet addiction” OR (adult[mesh] NOT (child[mesh] OR infant[mesh] OR “child, preschool”[mesh] OR "adolescent"[mesh])) OR Comment[sb] OR Letter[pt] OR Editorial[pt] OR (animals[mesh] NOT humans[mesh]) OR rat[ti] OR rats[ti] OR mouse[ti] OR mice[ti] OR “Acoustic Stimulation”[mesh] OR "Genetic Predisposition to Disease"[MAJR] OR "Gene Frequency"[MeSH] OR "Polymorphism, Single Nucleotide"[MAJR] OR genetics[sh] OR "athlete" OR "athletes" OR "Special Olympics" OR “coloring agents”[mesh])

*Appendix 2: Final coding sheet per study*

**Coding Form: Effects of Creative Movement Interventions on Individuals with Autism Spectrum Disorder: A Systematic Review**

Overall comments:

Coder: ____________________

Date of coding: ______________________

**Study characteristics:**

1. Study identifier (one string): 3 letters of 1^st^ author, 4-letters for year, 2001, : _
2. Author Names: ________________________________________________________
3. Year of publication: __________________ (Consider missing if unpublished work)
4. Full citation of publication (APA style): _______________________________________

________________________________________________________________________

________________________________________________________________________________________________________________________________________________

1. Source of study: ________ (1= journal, 2 = conference paper)
2. Location of study: ___ _____________
3. Year of data collection __ _____________

**Sample characteristics:**

1. Total sample size: __________
2. Age of subjects: ___ ______ (Mean (SD) and range)
3. Diagnosis of subjects: ____________________((0=Autism Spectrum Disorder (ASD), 1= Autistic Disorder, 2=Aspergers syndrome (AS), 3=Pervasive Developmental Disorder- Not Otherwise Specified (PDD-NOS), 4=Pervasive Developmental Disorder (PDD), 5 = Other(specify))
4. Measures used to establish diagnosis:

(0= standardized tests used such as Gilliam Autism Rating Scale (GARS), Childhood Autism Rating Scale (CARS), Social Communication Questionnaire (SCQ), Autism Diagnostic Interview (ADI-R), Autism Diagnostic Observation Schedule (ADOS), 1 = Physician report using DSM or ICD criteria 2= unstandardized observational measure, 3 = parent report/questionnaire, 4= Not mentioned)

_________________________________________________________________________________________________________________________________________________________________________________________________________________________________

1. Gender of subjects (# of males & females): _____________________________________
2. 6a: Type of subjects included (0=High/1=Moderate/2=Low functioning): ________
   1. 6b (0=High Verbal (HV) - phrases or sentences), 1=Low-verbal (LV)(few words), 2=Non-verbal (no words)): _____________

No information_____________________

1. IQ of subjects: ___________________(Mean (SD) and range)
2. Final # of subjects who participated in the study (Post attrition): ________

_________________________________

1. Socioeconomic status (Hollingshead scale): ____ _________________________________________________
2. Race (White, African America, Asian, etc.): _______________________________________________________________
3. Inclusion criteria: _________________________________________________________
4. Exclusion criteria: _________________

**Methodological quality of studies:**

1. Study design: ___________________________________________________________________ (0=Randomized controlled trial includes more than 1 study group and subjects randomly assigned to one of 2 or more groups, 1=Controlled clinical trials (clinical trial with more than one study group but no random assignment of subjects), 2=Pre-post designs that involve only 1 study group, 3=cross-over designs, 4= any other)
2. Study design (1 = Between-subjects design, 2 = Within-subjects design): _______________________________________________________________________
3. PEDro score for methodological quality: ______________________________________

Rating of 0-10. See pdf.

1. Type of control group used: _______________________

1 = random assignment of individuals

2 = matching individuals on some variable (specify variable: _____ _______________) and then random assignment

3 = tried to ensure some comparability of the non-equivalent control group for example: matching on some variable (specify variable:__________________________

4 = non-equivalence of comparison group was not addressed

5 = no control group (pre-post design)

1. Follow-up (1= present & #, 2 = absent, or Not mentioned): ______________________________
2. Intervals between follow ups: _______________________
3. Checks on treatment fidelity/integrity (Was there some way that they ensured that all trainers delivered the intervention the same way, in the case of group studies): 2= Very specific fidelity checklists prepared, 1=Some details provided, 0=No details provided: __________________________________________________________

**Experimental group characteristics:**

1. # of participants: _______________________
2. Total # who completed the study (after attrition): ____________________
3. Gender (# of males & females): ___________________
4. Age of participants (mean (SD) in years: _____________
5. Frequency of intervention (# of sessions/week): ___________________________
6. Time of intervention in minutes (per session): _____________________
7. Duration of intervention (days/weeks/months): _____________
8. Type of intervention: ___________________________________

(1= Active music therapy, 2 = Dance, 3 = Yoga/Mindfulness, 4 = Karate/Martial arts/Kata, 5 = Theater, 6 = Other including a combination of multiple components (please specify))

1. Setting of intervention (0 = at home, 1 = in child’s own school/autism center, 2 = indoor settings outside home like community center/YMCA/or any other indoor setting, 4 = other settings, please specify): _____________________________________________________________________
2. Components of intervention (provide short description of components of intervention):

________________________________________________________________________________________________________________________________________________________________________________________________________________________

1. Intervention design (0=Individual sessions/1=group therapy/2=unclear): ___________________
2. Intervention provider (all that apply):

(0=Occupational therapist/Physical therapist/Speech therapist, 1=Specialized instructor/therapist (music therapist, certified dance/yoga instructor, Sensei for martial arts, etc.), 2= Parent/caregiver, 3 = school teacher, 4 = Other, please specify)

(Specify additional training received by provider – for example, if researchers provided additional autism-specific training in the form of a workshop to martial arts instructors in the study): _________________________________________________

1. Strategies used during training sessions to ensure learning and compliance of children with training (0 = clear description of strategies used, 1 = some brief discussion/mention but sufficient details not provided, 2= no discussion) (list specific strategies used, for example, use of visual cues, Prompting schedule, reinforcement and prompting, modeling practice, part-whole practice, etc.)

________________________________________________________________________________________________________________________________________________

1. Progression of training activities (0= Discussed criteria to decide progression of training activities, 1= mentioned progression but did not outline specific principles of progressing training activities, 2 = not mentioned) If 0 mention, criteria for progression of training activities

________________________________________________________________________

1. Previous relevant experience of subjects in same activity in months (0 – Information provided, 1= No information provided)

(even if information is specified in years, convert to months): __________________

Specify any other relevant details of experience (type, setting, etc.)__________________________________________________________________________________________________________________________________________

1. Pre-requisites of the specific intervention (if any) (for example, if it is an advanced karate/dance program, then completion of a basic training program would be considered a pre-requisite for the advanced training): ________________________________________________________________________________________________________________________________________________
2. Other therapies subjects were receiving at the same time for example, PT, OT, Speech therapy, etc. (specify): Along with details of Frequency, Intensity, Time, and Type - Provide details of when the child is receiving this therapy

**Control group characteristics:**

1. # of control groups: __ _______________
2. # of participants in each control group: ________________________
3. Age of participants (Mean (SD) in years: ______________
4. Total # who completed the study (after attrition) in each control group: _____________________________________________________________
5. Gender (# of males & females) in each control group: __________________________
6. Frequency of intervention: ___________________________________________
7. Time of intervention (per session): _______________________________________
8. Duration of intervention (days/weeks/months): _____________________________
9. Type of intervention in control groups: ________________________________________________________________________________________________________________________________________________
10. Progression of training activities (0= Discussed criteria to decide progression of training activities, 1= mentioned progression but did not outline specific principles of progressing training activities, 2 = not mentioned) If 0 mention, criteria for progression of training activities

________________________________________________________________________

1. Other interventions that the control group is receiving if any (if mentioned in the paper)

**Dependent variables:**

1. Type of dependent variable: __

(1 = Social measures example: joint attention behaviors, turn taking, eye contact

2 = Communication skills such as verbal and non-verbal skills (gestures))

3 = Behavioral problems and affective/emotional states– stereotypical behaviors, aggression, self-injurious behaviors, negative behaviors, negative affect, anxiety, mood, etc.

4 = Sensory problems like hyper-responsiveness to sensory stimuli, negative responses to certain types of sensory inputs, seeking vestibular sensation, oral hypersensitivity, peering at objects, etc.

5 = Motor behaviors like balance, coordination, fine motor skills, gross motor skills like jumping, skipping, etc.

6 = Quality of life outcomes

7 = Self-care skills and activities of daily living

8 = Cognitive skills (some studies have looked at executive function)

10 = Others (specify______________________________)

1. Variables analyzed (Duration or Rate or Frequency of something or score on an assessment, etc.) ___________________________________________________ _
2. Instrument/s used for assessment of dependent variable (if multiple dependent variables, mention all different scales used): _________________________________________________________________
3. Type of scale/measure used for assessing dependent variables:

(1 = Standardized scale (has norm referenced values; also has a component of expert rating), 2 = observational measure, 3 = video/audio coding, 4 = questionnaires/interview, 5 = others like computerized tasks, etc. (specify____________________________________________), 6 (Not mentioned)

______ _________________________________________________________

1. Reliability and validity of scales used (did the authors report reliability/validity of assessments or scales used in the study? 0 = Mentioned and values provided, 1= Mentioned but no specific values provided, 2= Not mentioned) – Mark scores for reliability and validity of assessments

*For reliability*, the kind of concepts that you are looking for are inter-rater reliability, intra-rater reliability, test-retest reliability, internal consistency

*For validity*, look for terms such as face validity, content validity, convergent validity, discriminant validity, criterion-related validity, concurrent validity: __________________________________

1. Reliability of assessments used (that is did researchers establish inter- and intra-rater reliability for the coded outcome measure): (1= not mentioned, 0 = reported) ___ __
2. Time-point of measurement of dependent variables (that is whenever the testing measured administered, for example, at pretest, at posttest, etc.): ________________
3. # of dependent variables for which effect sizes will be obtained from the study: ________________________________
4. # of ES obtained from the study: _______________________________________
5. Direction of effect (1 = positive/improvement in outcome, 2 = trend towards significance, 3 = negative/worsening of outcome, 4 = no effect/no change) per DV: __________________
6. Summary statistic used in the paper per dependent variable (DV):

4 = Beta or B coefficients with their standard errors from regression models

3= Mean, SD, SE, MS Error of the variable

2=Proportion of a certain behavior and how it changed, Ordinal/Nominal Scores

1=Figures with/without units.

0=No data, just qualitative descriptions of change. No numbers provided.

(These scores should be mutually exclusive. Summation should not be necessary)

1. Type of statistic used per DV (brief description of statistics per dependent variable): __________________________________________________________________________________________________________________________________________

8= Any other analyses (please specify_________________________)

7= Regressions (linear regression, multiple regression, hierarchical linear regressions, etc.)

6=Effect Sizes using eta-squared, Cohen’s d, R^2^ or other effect size statistic

5 = Multivariate statistics (MANOVA, MANCOVA)

4=ANOVA, ANCOVA

3=t-test, Z test

2=Non-parametric statistics like chi-square tests to compare ordinal/nominal scores of change with training, Kruskal wallis, Wilcoxon test

1=correlations for reliability of measures

1. Some search terms were appropriately modified for other data bases. [↑](#footnote-ref-1)
